# Seasons of *Syn*

**DOI:** 10.1002/lno.11374

**Published:** 2019-11-19

**Authors:** Kristen R. Hunter‐Cevera, Michael G. Neubert, Robert J. Olson, Alexi Shalapyonok, Andrew R. Solow, Heidi M. Sosik

**Affiliations:** ^1^ Josephine Bay Paul Center Marine Biological Laboratory Woods Hole Massachusetts; ^2^ Biology Department Woods Hole Oceanographic Institution Woods Hole Massachusetts; ^3^ Marine Policy Center Woods Hole Oceanographic Institution Woods Hole Massachusetts

## Abstract

*Synechococcus* is a widespread and important marine primary producer. Time series provide critical information for identifying and understanding the factors that determine abundance patterns. Here, we present the results of analysis of a 16‐yr hourly time series of *Synechococcus* at the Martha's Vineyard Coastal Observatory, obtained with an automated, in situ flow cytometer. We focus on understanding seasonal abundance patterns by examining relationships between cell division rate, loss rate, cellular properties (e.g., cell volume, phycoerythrin fluorescence), and environmental variables (e.g., temperature, light). We find that the drivers of cell division vary with season; cells are temperature‐limited in winter and spring, but light‐limited in the fall. Losses to the population also vary with season. Our results lead to testable hypotheses about *Synechococcus* ecophysiology and a working framework for understanding the seasonal controls of *Synechococcus* cell abundance in a temperate coastal system.

Shall I compare thee to a summer's day?

Thou art more lovely and more temperate.

Rough winds do shake the darling buds of May,

And summer's lease hath all too short a date.

Sometimes too hot the eye of heaven shines,

And often is his complexion dimmed;

And every fair from fair sometime declines,

By chance, or nature's changing course, untrimmed;

—William Shakespeare, Sonnet 18

For many multicellular organisms, daily variability in environmental conditions (captured so eloquently by Shakespeare) affects their vital rates less than does variability at seasonal or annual scales. For a single‐celled marine phytoplankter, however, a summer's day is a long time; we know that how quickly a cell grows and divides are influenced (if not determined) by whether “too hot the eye of heaven shines” or “his complexion [is] dimmed.” Understanding seasonal and long‐term phenomena therefore requires knowledge of daily dynamics. Here, we report on our ongoing, 16‐yr‐long study to understand how “nature's changing course” determines the population dynamics of “*Syn*”—the picocyanobacterium *Synechococcus*—in each season over an annual cycle.

Marine picocyanobacteria are numerically dominant in both open ocean and coastal waters and are estimated to be responsible for up to a quarter of marine primary production (Flombaum et al. 2013). Ever since their relatively recent discovery (late 1970s for *Synechococcus* (Waterbury et al. 1979) and mid‐1980s for *Prochlorococcus* (Chisholm et al. 1988)), researchers have sought to understand their distributions over time and space. Field observations, in situ experiments, and ecophysiology studies have yielded important insights into the factors, both environmental and biological, that affect the abundance of these organisms.

*Prochlorococcus* is found primarily in tropical and subtropical oligotrophic regions, extending throughout the entire photic zone, favoring warmer (> 15°C) waters (Olson et al. 1990a; Johnson et al. 2006). In contrast, *Synechococcus* is found over a wider geographic range, extending from open ocean to coastal regions and up to high‐latitude polar areas (Olson et al. 1990b; Zwirglmaier et al. 2007; Paulsen et al. 2016). This group can tolerate cooler and more nutrient rich waters. For *Synechococcus*, temperature is believed to be one of the main factors determining population growth. Strong relationships have been observed between cell abundance and division rate and temperature, especially in temperate regions (Waterbury et al. 1986; Agawin et al. 1998; Li 1998; Li and Dickie 2001; Tsai et al. 2008). Biological loss from a *Synechococcus* population can come from protist grazers (nanoflagellates, ciliates, dinoflagellates) or viral lysis. Depending on the season and location, both agents have been observed to control *Synechococcus* abundances (Agawin et al. 1998; Baudoux et al. 2008; Tsai et al. 2008).

Time series data are invaluable for exploring the factors that determine abundance. Multiyear observations allow for separation of subseasonal, seasonal, interannual, and decadal variability. Dynamics in the plankton can change quickly though, on the order of hours to days, and observations are needed at timescales that match cells' physiology and ecology. This observational challenge has been met over the past ~ 20 years with the development of automated flow cytometers (Dubelaar et al. 1999; Olson et al. 2003; Swalwell et al. 2011) and imaging flow cytometers (Sieracki et al. 1998; Olson and Sosik 2007). These instruments have provided high temporal resolution and species‐specific observations for characterizing phytoplankton dynamics. At the Martha's Vineyard Coastal Observatory (MVCO), located on the Northeast U.S. Shelf (now a U.S. long‐term ecological research network site, NES‐LTER), the automated flow cytometer FlowCytobot (FCB; Olson et al. 2003) has been deployed since 2003, with year‐round deployments beginning in 2007, providing hourly observations of the picophytoplankton community.

This high resolution time series has enabled new insights into the *Synechococcus* population at MVCO. At this location, *Synechococcus* is often the numerically dominant phytoplankter, and can be an important contributor to coastal primary production (Li 1994; Jardillier et al. 2010). Measurements from FCB show that *Synechococcus* exhibits a dramatic spring bloom, increasing in cell concentration from a few hundred cells mL^−1^ up to ∼ 10^5^ cells mL^−1^ in the span of a few months. Flow cytometry (FCM) is especially powerful because this technique enables not only cell counts, but also observations of individual cell properties (in particular, cell volume) that can indicate the physiological status of the population. By using such observations in conjunction with a matrix population model, we have been able to accurately estimate the daily population division rate of *Synechococcus* in laboratory cultures and in the field (Hunter‐Cevera et al. 2014, 2016a). Division rate is a critical metric for understanding how the environment controls cell growth and its contribution to changes in cell concentration. In previous work, we analyzed relationships between division rate and environmental variables to show that the underlying trigger of the spring bloom at MVCO is the seasonal increase in water temperature (Hunter‐Cevera et al. 2016a) and that losses to the population strongly modulate the bloom's trajectory.

In this article, we extend the exploration of drivers of *Synechococcus* seasonal cell abundance patterns obtained with FCB at MVCO for the entire annual cycle. We examine how cell abundance, division and loss rates, and cellular properties are related to environmental variables and how these relationships change seasonally. We find that drivers of population growth, and the balance between growth and loss are not the same over the annual cycle. Combined with insights from studies of *Synechococcus* physiology and ecology, we present a synoptic framework for understanding seasonal cell abundance changes in temperate regions.

## *Materials and methods*

### Study site and sampling

**Figure 1:**
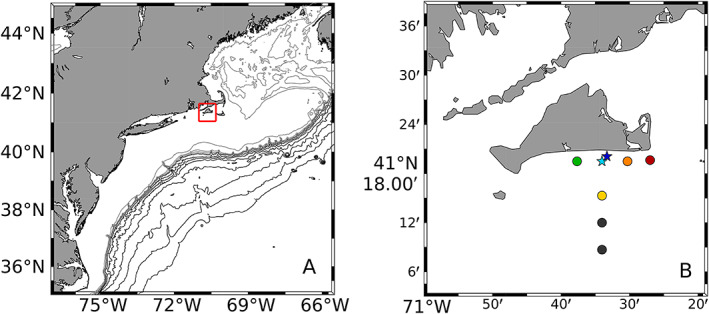
(**A**) Location of MVCO on the northeast U.S. continental shelf. Light contours indicate depths of 100–400 m, spaced every 100 m. Black contours indicate depths from 500 to 4000 m, spaced 500 m. Red box indicates area shown in (**B**). (**B**) Points are locations of radiometer deployments, corresponding with Fig. 2. MVCO tower location is indicated by cyan star, node by blue star. Southernmost gray points were sampled, but not used in calculation of attenuation coefficients.

MVCO is a cabled facility that consists of a shore‐based station and meteorological mast, an undersea node located at 12 m depth, and a tower structure in 15 m water, rising 10 m above sea level. The node is located 1.5 km south of the island of Martha's Vineyard, MA (41°20.19′N 70°33.38′W) and the tower is located 3 km offshore (41°19.5′N 70°34.0′W, Fig. 1). Mean sampling depth of FCB is ∼ 4.2 m, with daily tidal excursions of ∼ 1 m. From 2003 to 2004, FCB was located at the bottom, and sample delivery occurred through an integrated pump and Tygon line with inlet. Beginning in 2005, FCB was deployed on the tower beam. Deployments from 2003 to 2007 were conducted with only one instrument, but after 2007, with the availability of a second FCB, data have been obtained from alternating deployments of the instruments. Core measurements at the MVCO facility include a range of meteorological and hydrographic data. Measurements of incident radiation are made at the meteorological mast with an Eppley pyranometer and 20‐min resolution data were downloaded from ftp://mvcodata.whoi.edu/pub/mvcodata/. For years 2005–2007 and 2010–2013, if light data were unavailable, data gaps were filled with radiation measurements obtained from a NOAA National Buoy Data Center buoy (Sta. 44008), located southeast of Nantucket (40°30′9″N 69°14′48″W, ∼ 144 km from MVCO) (buoy data are unavailable for other years). If radiation data were unavailable from either source, then day was omitted from our analyses. Daily solar radiation was calculated by integrating incident radiation over 24 h. Spectral quality and quantity of light at depth were measured on 15 separate days during 2007–2012 at the tower, 12‐m node, and other nearby locations with a radiometer (SeaBird Electronics HyperPro II, *see* Fig. 1 for locations).

Temperature was measured continuously at ∼ 4 m at the tower with a MicroCat CTD (Seabird Electronics). Data gaps in the temperature record were filled with regression‐adjusted temperature measurements taken at the 12‐m node (downloaded from ftp://mvcodata.whoi.edu/pub/mvcodata). Linear regressions used in the correction were calculated for each month. The records from the tower and 12‐m node have been previously shown to be very similar (Peacock et al. 2014). For any remaining data gaps, temperature data from CTD casts taken during sampling trips to MVCO were also used. A total of 104 CTD casts were available, taken between 2007 and 2016 aboard the R/V *Tioga*. Profiles were collected without regard to tidal cycle. Only the downcast portion of casts was used (manually identified) and data were averaged by depth in 0.2 m bins.

Near surface water samples were collected bimonthly‐to‐monthly for nutrient analysis. Samples were filtered through a 0.2 *μ*m Sterivex® filter into acid‐washed vials and frozen at −20°C. Phosphate, ammonium, silicate, and nitrate + nitrite concentrations were determined by standard autoanalyzer techniques at the Woods Hole Oceanographic Institution Nutrient Analytical Facility (Woods Hole, MA).

### Estimation of light exposure

To explore how light affects *Synechococcus* population dynamics, we need knowledge of the in situ light environment. The light environment that cells experience depends on incident solar radiation, attenuation in the water column, and mixing, as this affects the depths (and thus light levels) cells encounter. We estimated annual light exposure for the *Synechococcus* we measured at 4 m depth from (1) time series of incident solar radiation, (2) attenuation coefficients from available radiometer profiles, and (3) estimation of stratification at MVCO from continuous temperature records at two depths.

#### *Photosynthetically active radiation attenuation coefficients*

We calculated attenuation coefficients for photosynthetically active radiation (PAR) from measured profiles of spectral irradiance (*μ*W cm^−2^ nm^−1^) obtained from a total of 71 radiometer deployments conducted on 15 separate days from 2007 to 2012. Data processing was done with custom scripts in MATLAB (available at http://github.com/hsosik/NES‐LTER/tree/master/mvco_light_env). Briefly, closest‐in‐time dark measurements were subtracted from both surface reference and subsurface downwelling irradiance measurements. PAR (W cm^−2^) was calculated as the integral of irradiance from 400 to 700 nm. Attenuation coefficients were estimated assuming exponential attenuation with depth:

(1) $$E\left(z\right)=E\left(0^{-}\right)\exp\left(-K_{d}\cdot z\right),$$

where *K*_*d*_ is the attenuation coefficient, *z* is depth, and *E*(*z*) and *E*(0^−^) refer to irradiance at depth and just below the surface, respectively. A linear least squares routine in MATLAB (v2018b, regress) was used for *K*_*d*_ estmation: ln(*E*(*z*)) = *b* + (−*K*_*d*_ · *z*), where *b* was also fitted but should correspond to ln(*E*(0^−^)). *K*_*d*_ values were estimated for each cast within a deployment (often multiple casts) and then averaged within a deployment.

#### *Evaluation of water column stratification*

We constructed a stratification index from the difference between continuous temperature measurements at two depths (4 m at the tower station and 12 m at the node). We examined temperature and density profiles from CTD casts to help us evaluate and interpret this index.

From available profiles, we found that the temperature difference between 4 and 12 m (denoted as Δ*θ*(4, 12)) is correlated with the density difference between those depths (Δ*σ*(4, 12)) (Supporting Information Fig. S1A). We also found that Δ*σ*(4, 12) can provide an index of the density structure within the water column. Density profiles varied from well mixed to those containing distinct stratified layers. Stratification was identified following methods described in Brainerd and Gregg (1995) and Kara et al. (2000); if the variation in potential density with depth exceeded 0.2 kg m^−3^ from either a surface or 4‐m reference value, the profile was considered stratified. A 4‐m depth reference value was used when strong surface (< 3 m) stratification was observed. We classified observations as well‐mixed, profiles with a stratification feature below 3 m, or profiles that only contained surface stratification (within the top 3 m). A threshold value of 0.2 kg m^−3^ for Δ*σ*(4, 12) reliably identified profiles that contained stratification below 3 m (Supporting Information Fig. S1B). This value was associated with temperature difference of 0.68°C (based on linear regression between Δ*θ*(4, 12) and Δ*σ*(4, 12), Δ*θ* = −0.0354 − 3.24·Δ*σ*, *R* ^2^ = 0.807). As profiles were collected without regard to tidal cycle, we believe our stratification index should reflect a composite effect across various phases of the tide over the annual cycle.

We then determined when hourly temperature measurements from 4 and 12 m (from the tower and 12‐m node, respectively) exceeded a difference of 0.68°C, indicating likely stratification. We classified an entire day as stratified if greater than two‐thirds of daylight hours exceed this temperature difference. We find that for ∼ 97% of days, the water column is well mixed based on this criteria (Supporting Information Fig. S2). Notable stratification does occur for some days in summer and, interestingly, in winter.

#### *Average in situ light*

We estimated an annual climatology of light exposure that *Synechococcus* cells would experience at depth from the daily climatology of incident radiation and climatology of attenuation coefficients. We assumed a well‐mixed water column throughout the year, based on our stratification results. We do not have information about the timescale of mixing, and therefore make a simple assumption that cells pass through the entire water column in a day.

**Figure 2:**
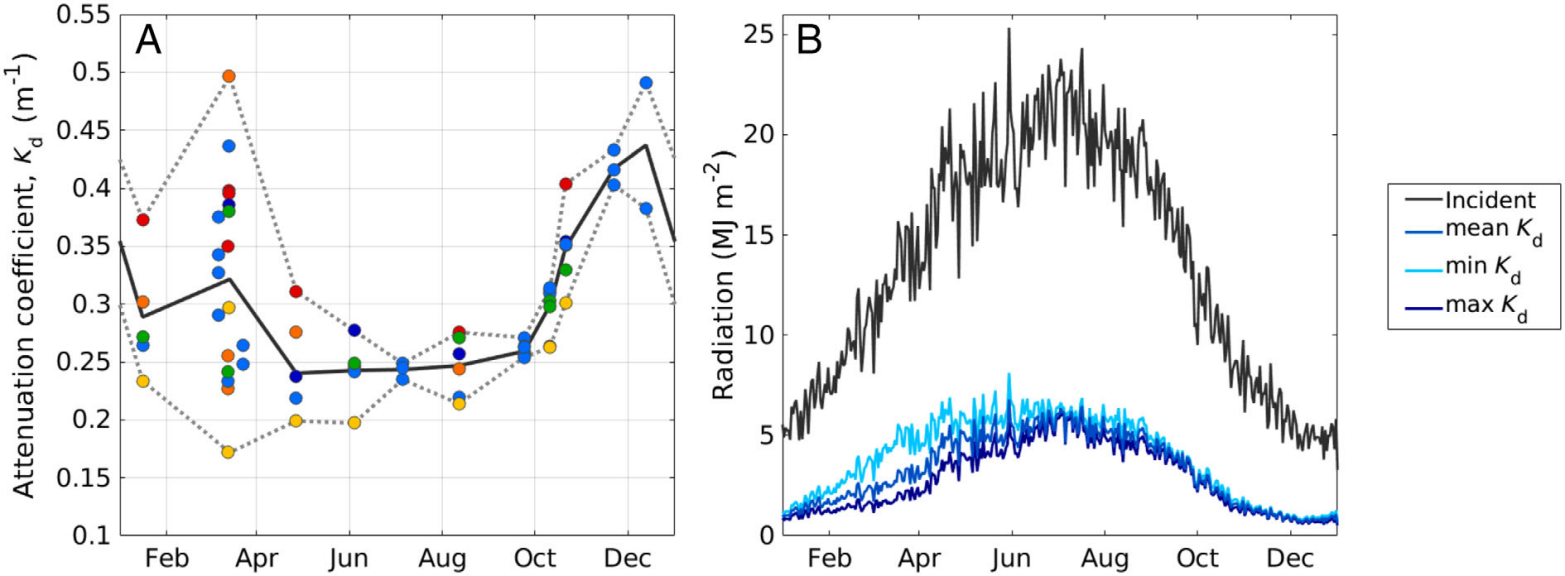
(**A**) Attenuation coefficients plotted against year day of measurement. Points are color‐coded based on deployment locations in Fig. 1. Solid gray line is mean of attenuation coefficients (*K*_*d*_) across locations and time as described in text. Dashed gray lines indicate range of *K*_*d*_ values. (**B**) Climatology of incident radiation (gray line) and attenuated radiation (blue lines) calculated from interpolated mean, minimum, or maximum *K*_*d*_ values (blue, light blue, and dark blue lines, respectively, see text for more details).

Attenuation coefficients were averaged across nearshore locations, excluding the two most southern sampling locations, as these were more characteristic of deeper shelf water. If the sampling year day occurred within 2 weeks of another sample, *K*_*d*_ values were further averaged together (Fig. 2A). We then linearly interpolated these averages through time to estimate a *K*_*d*_ value for each year day. For comparison, interpolation based on maximum and minimum observed *K*_*d*_ values was also performed (Fig. 2B).

From interpolated *K*_*d*_ values, and assuming cells experience all depths within a day, we calculated an average light exposure as:

(2) $$\bar{E_{d}}\left(t\right)=\frac{1}{15}\int_{15}^{0}\bar{E_{0}}\left(t\right)\cdot \exp\left(-\bar{K_{d}}\left(t\right)\cdot z\right)\;\mathrm{dz},$$

where $\bar{E_{0}}\left(t\right)$ is the climatological value of incident radiation on year day *t*, $\bar{K_{d}}\left(t\right)$ is the interpolated *K*_*d*_ value, and $\bar{E_{d}}\left(t\right)$ is the resulting average light level. Here, 15 refers to the average height in meters of the water column at MVCO.

### FlowCytobot

Details of the design and performance of the automated, submersible flow cytometer, FCB, are described elsewhere (Olson et al. 2003). Briefly, the instrument uses a 532‐nm solid‐state laser for excitation and is able to detect individual cell forward and side light scattering and fluorescence at 575 and 680 nm. FCB includes pairs of linear amplifiers set to different gains to extend the dynamic range, signal processing electronics, and a data logger for instrument control and data acquisition. Samples are analyzed in 0.25 mL syringe volumes, and are analyzed at rates of either 5, 10, or 20 min per syringe depending on time of year (rate is set manually to keep events per second in reasonable range). Data analysis and enumeration of *Synechococcus* cells were as described in Sosik et al. (2003) (available at http://github.com/hsosik/NES-LTER/tree/master/fcb_processing). *Synechococcus* were discriminated from other phytoplankton and particles by their orange fluorescence from phycoerythrin (PE), red fluorescence from chlorophyll, and small light scattering signals. Polystyrene microspheres (beads) (Polysciences) of diameter 0.5 *μ*m (polychromatic) and 1.0 *μ*m (red‐fluorescing) were measured automatically as reference particles every ∼ 17 h during deployments. Cellular scattering and PE fluorescence values were normalized with 1.0 *μ*m bead values for analysis (on corresponding channels). Right angle scattering has been calibrated to estimate cell volume (Olson et al. 2003). Note that while we collect red fluorescence data from *Synechococcus*, we do not include it in the below analysis. PE emissions extend and spill over into the red channel due to an asymmetric long‐wavelength shoulder. Hence, red fluorescence data are difficult to interpret in terms of physiological or photosynthetic properties.

### Division rate estimation

Division rates were estimated with the matrix population model and estimation techniques described in Hunter‐Cevera et al. (2016a) (with code now moved to http://github.com/hsosik/NES-LTER/tree/master/phyto-division-rate-model). The model represents diel changes in cell size distributions of *Synechococcus*. Cells are binned into discrete size (volume) classes and within a model time step (10 min), cells may grow into the next size class, divide into a smaller size class, or remain in the same class. In the model, cell growth depends on daily incident radiation, and cell division depends on size class. We fit the starting cell size distributions for each subpopulation to log normal distributions. We fit the model to hourly observations of cell sizes by the method of maximum likelihood, assuming that the observed counts in different size bins follows a multinomial‐Dirichlet distribution. From the best fitting model, we calculate a division rate from the starting and ending numbers of model cells. Importantly, model estimates do not rely on observed changes in the concentration of cells, only changes in the distribution of cell sizes. Furthermore, grazing of *Synechococcus* does not appear to affect cell size distributions (Hunter‐Cevera et al. 2014), such that these division rate estimates are not affected by this process.

### Net growth rate, loss rate, and other calculations

We calculated population net growth rate, *μ*_net_(*t*), from smoothed cell concentration data (48‐h running mean to reduce tidal and other high frequency effects), as follows:

(3) $$\mu_{\mathrm{net}}\left(t\right)=\ln\frac{\bar{N}\left(\tau \left(t\right)+24\right)}{\bar{N}\left(\tau \left(t\right)\right)},$$

where *τ*(*t*) is the dawn hour of each day, *t*, and $\bar{N}\left(\tau \left(t\right)\right)=\frac{1}{3}\sum_{j=-1}^{j=1}N\left(\tau \left(t\right)+j\right),$ where *N*(·) is the hourly smoothed cell concentration. $\bar{N}\left(\tau \left(t\right)\right)$ gives an average cell concentration in the 3‐h interval surrounding dawn to avoid spuriously high or low net growth rates based on choice of hour. The time period for calculating net growth rate matches the time period used for division rate estimation. Loss rates were calculated by subtracting net growth rate from division rate for each day available in the data set and reflect combined losses from predation (grazer ingestion and viral lysis) and the net effect of cell emigration and immigration from advection.

With the exception of nutrient concentrations, daily‐resolved mean annual patterns (i.e., climatologies) were calculated by averaging values for each year day across available years. Weekly patterns were constructed by taking the median of all days belonging to a given week for all years available in the data set (i.e., daily climatology values were not used to form weekly values). Anomalies were constructed by subtracting the average year day value from daily data.

Construction of climatologies for cell volume and PE fluorescence must take into consideration that both measurements typically display diel patterns. Since we measure the volume and fluorescence of each cell, we can construct distributions of those properties for the population with hourly resolution. The modes of those distributions effectively track diel changes. To examine lower frequency changes (e.g., seasonal to annual patterns) independent of diel fluctuations, we chose to consider the daily minimum value of the population mode for cell volume and the population mode value at dawn for PE fluorescence. Investigation of minimum cell volume should reflect intrinsic changes in cell size, whereas a daily average or maximum value would be influenced by division rate. For PE fluorescence, values at dawn avoid interpretation challenges that come with daily minimum, average, or maximum values. Low fluorescence values can result from cell division or fluorescence quenching during the day, and maximum values result from pigment production or fluorescence decoupling (Vaulot and Marie 1999; Jacquet et al. 2001). We also investigate dawn PE fluorescence normalized to cell volume to remove the effect of cell size. For the remainder of the text, cell volume refers to the minimum mode and PE fluorescence refers to the mode at dawn for each day in the data set.

We construct only monthly climatologies of nutrient concentrations as these measurements are sparse. Climatologies were calculated as median values for all year days belonging to each month.

### Curve fitting of division rate with radiation

Saturating responses of division rate to incident radiation were estimated by fitting observations to the following functional form:

(4) $$\mu =\mu_{\max}\left[1-\exp\left(\frac{-\mathrm{\alpha E}}{\mu_{\max}}\right)\right],$$

where *μ* is daily estimated division rate, *E* is observed daily incident radiation, and *α* and *μ*_max_ are shape parameters estimated with a nonlinear least squares optimization offered in MATLAB (v2018b, lsqnonlin). To explore interacting effects of light and temperature, the light dependence of *μ* was evaluated separately for observations binned by temperature in 2°C intervals from 0°C to 22°C.

## *Results*

At MVCO, we find that many of the environmental variables and properties of the *Synechococcus* population display strong seasonality. We describe those patterns below and explore relationships between them in the discussion.

### Environmental conditions

**Figure 3:**
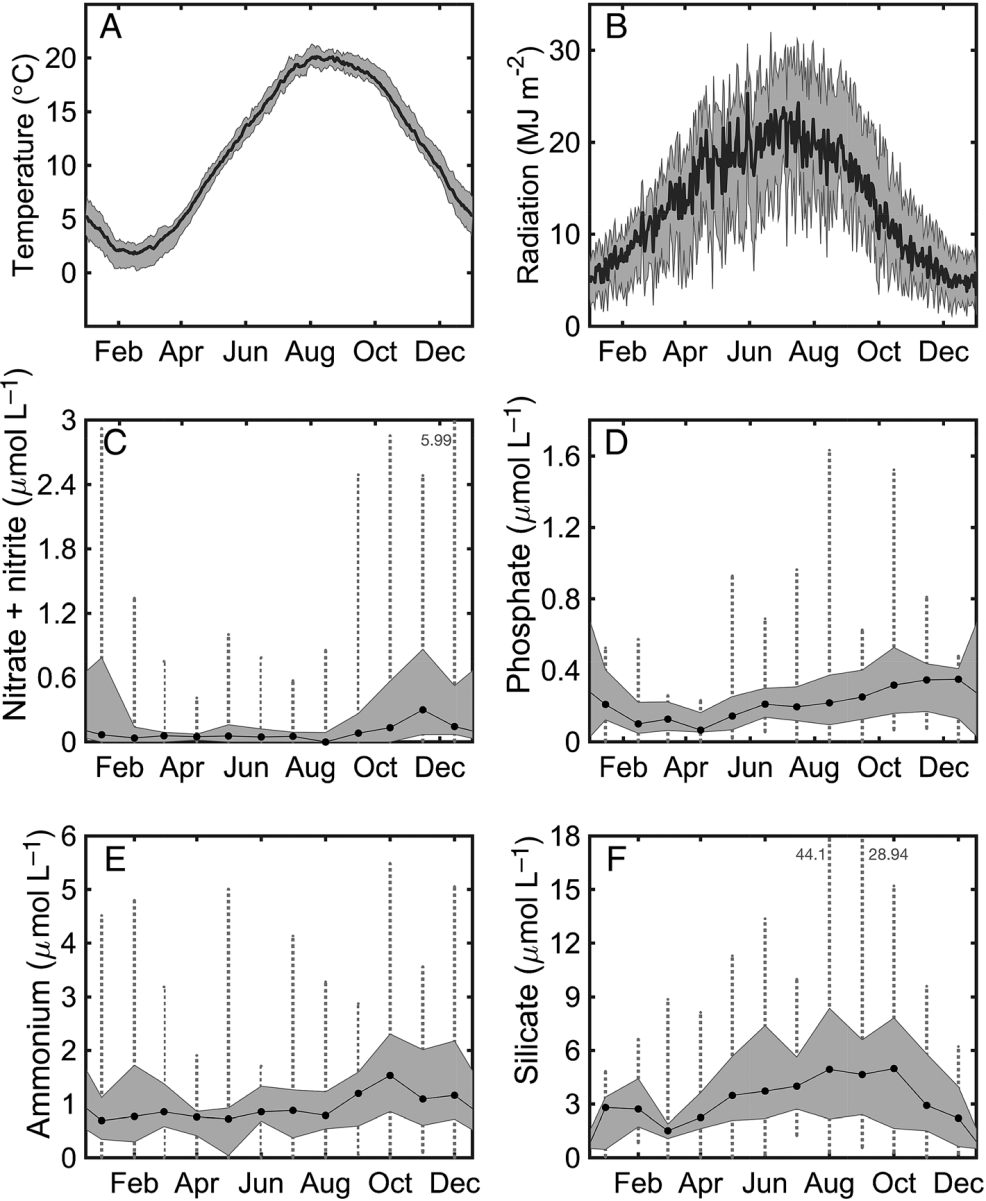
Daily climatology of (**A**) temperature (black line) and (**B**) radiation (black line) from 2003 to 2018. Shaded gray areas indicate one standard deviation from daily mean. Median concentrations of (**C**) nitrate + nitrite, (**D**) phosphate, (**E**) ammonium, and (**F**) silicate. For panels (**C**–**F**), the gray shaded area encompasses the 25^th^ to 75^th^ quantiles. Dotted vertical lines indicate the range of data observed for each month. If the value is outside the panel limits, the value is indicated in gray text.

As expected for a temperate location, water temperature exhibited a wide range with annual minima of −2°C to 2°C in mid‐February and maxima of 20°C to 22°C in mid‐August (Fig. 3A). Daily maximum solar radiation dropped below 5 MJ m^−2^ in late December and exceeded 25 MJ m^−2^ in late June (Fig. 3B). Attenuation coefficients for PAR followed a seasonal pattern with lowest values in late spring and summer, and highest values in the fall, winter, and early spring, although spring is highly variable (Fig. 2A). As expected for a coastal location, highest irradiance values were located in the green wavelengths, with maximum energy found at 535 nm or between 550 and 560 nm, but occasionally at bluer wavelengths of ∼ 495 nm (data not shown). Spectral distributions did not seem to follow an annual pattern.

Measured nutrients (nitrate + nitrite, ammonium, inorganic phosphate, and silicate) displayed some seasonality, though in contrast to temperature and light, the amplitude of sample variability exceeded any seasonal variability (Fig. 3C–F). Nitrate + nitrite concentrations were typically low (≤ 1 *μ*mol L^−1^), and ∼ 30% of samples were below the limit of detection of 0.04–0.05 *μ*mol L^−1^. Higher values were found in fall and early winter. Ammonium concentration was usually ≤ 3 *μ*mol L^−1^ and inorganic phosphate ≤ 1 *μ*mol L^−1^. Silicate concentration was typically higher in late summer to early fall months and was usually between 3 and 9 *μ*mol L^−1^.

### Cell concentration

**Figure 4:**
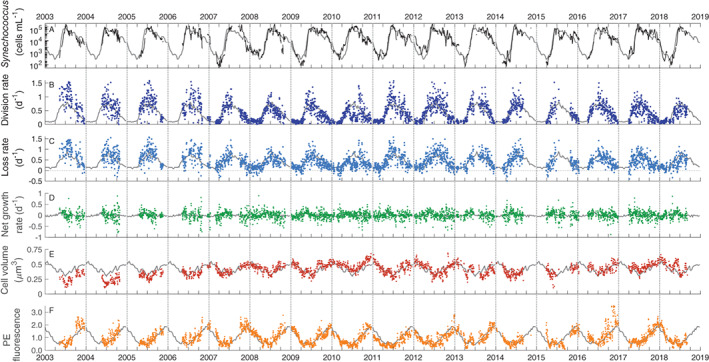
Daily time series of *Synechococcus* properties for (**A**) cell concentration, log scale, (**B**) daily division rate, (**C**) calculated daily loss rate, (**D**) net growth rate, (**E**) cell volume, and (**F**) cellular PE fluorescence. Gray lines in each panel are annual patterns (weekly median climatology) for reference.

**Figure 5:**
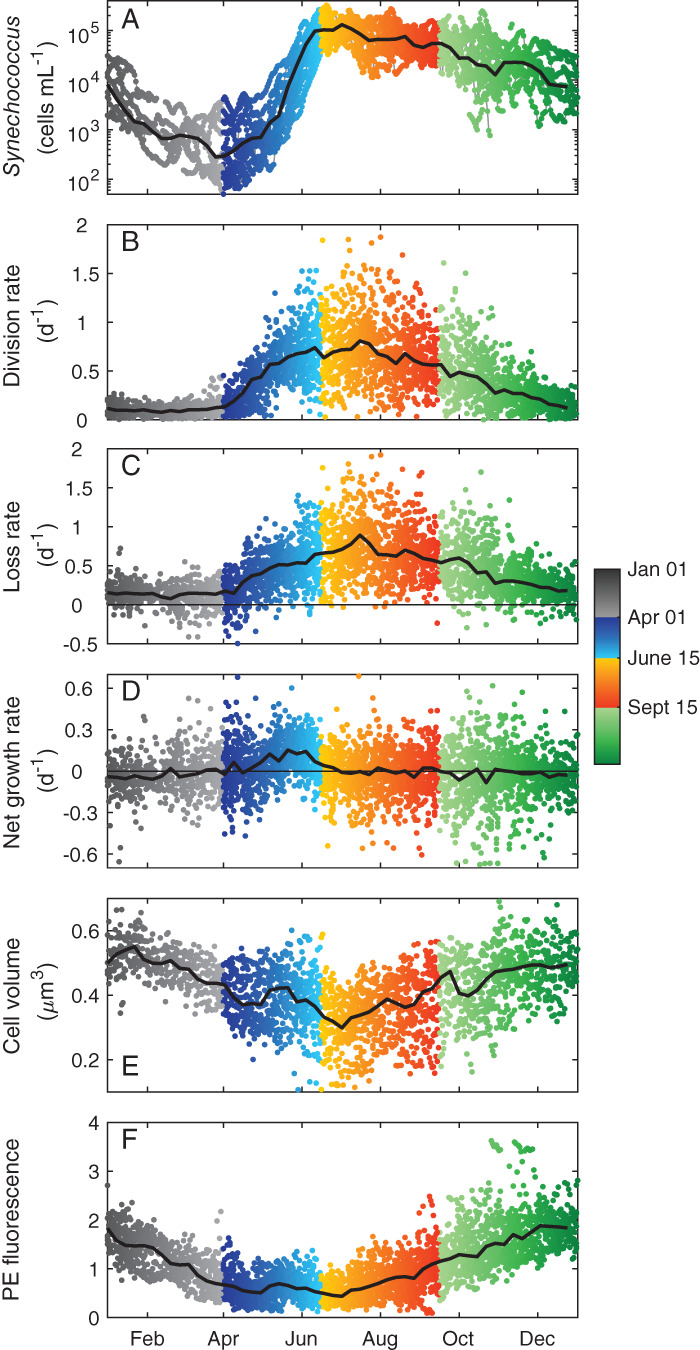
Scatter plots of (**A**) *Synechococcus* concentration, (**B**) division rate, (**C**) loss rate, (**D**) net growth rate, (**E**) cell volume, and (**F**) cellular PE fluorescence by year day for data from 2003 to 2018. Black line in each panel is weekly median climatology. Color indicates season and year day.

*Synechococcus* cell concentration and dynamics for the period 2003 to mid‐2016 had been previously reported in Hunter‐Cevera et al. (2016a). The addition of data through the fall of 2018 reinforced the general patterns we observed in the 2003–2016 data. *Synechococcus* cell concentration is strongly seasonal (Figs. 4A, 5A, Supporting Information Figs. S3A–S6A). Cell abundance from late winter through mid‐spring (February–April) was low, between 100 and 1000 cells mL^−1^ (Supporting Information Fig. S10). A systematic increase in cell abundance (2–3 orders of magnitude) from April through mid‐June characterized the spring bloom (Supporting Information Fig. S7). Cell concentration reached annual maximum values (∼ 2 × 10^5^ cells mL^−1^) typically in mid‐June, but higher values were observed later in the summer for some years (i.e., 2008, 2010; Supporting Information Fig. S8). From mid‐summer into early winter (July–December), cell concentration exhibited a slow decline, but remained relatively high (above 10^4^ cells mL^−1^, Supporting Information Fig. S9). Superimposed on this decline were fluctuations (up to ∼ 10^4^ cells mL^−1^) that occurred on time scales of a few weeks. These shorter duration changes were observed throughout the annual cycle, but were most pronounced during summer and early fall. Beginning in January, cell concentrations typically exhibited a more rapid decline to annual minimum values.

### Division, net growth, and loss rate

Cell division rate is also strongly seasonal (Figs. 4B, 5B, Supporting Information Figs. S3B–S6B). Division rate was consistently low (∼ 0.1 d^−1^) in January and February. In March, the rate began to increase, peeking in June (∼ 0.9 d^−1^). The division rates generally remained high (> 0.7 d^−1^) in the summer, but could be highly variable with some periods of low to moderate rates (0.25–0.5 d^−1^) during the summer. Division rates typically declined from July through October, with a sharper decline beginning in late October or early November. During this sharper decline, division rates decreased to wintertime low values by January.

Most values of daily net growth rate fall between −0.25 and 0.25 d^−1^ over the entire time series (Figs. 4D, 5D, Supporting Information Figs. S3D–S6D). On average, net growth rate hovered near zero for most of the year, but with systematic imbalances during the spring bloom and fall decline. As determined from back‐calculation, loss rates followed a seasonal pattern very close to that of division rate. Losses closely tracked division rate in magnitude over the annual cycle (Figs. 4C, 5C, Supporting Information Figs. S3C–S6C), but with slight offsets depending on the season (Fig. 10, top panels), such that values were generally lower than division rates in spring but slightly higher during the fall.

### Cell properties

Cell properties also displayed seasonal patterns. Cell volume was highest during winter months (Figs. 4E, 5E), with largest values observed in January (∼ 0.6 *μ*m^3^), and smallest values in summer, usually in July (∼ 0.25 *μ*m^3^) (see also Supporting Information Figs. S3E–S6E). Note that the annual change in minimum volume was generally larger than the majority of diel volume changes (Supporting Information Fig. S13). Cellular PE fluorescence was typically highest between October and January and lowest during May–July (Figs. 4F, 5F, Supporting Information Figs. S3–S6F).

## *Seasons of* Syn

We continue our investigation of the strikingly repeatable seasonal cycles of *Synechococcus* cell abundance from a 16‐yr time series of hourly FCM measurements at MVCO. To help explain these cycles, we consider a variety of population measurements (estimates of daily division rate, calculated loss rate, cell volume, and cellular PE fluorescence) and concurrent environment variables (water temperature, incident light, and nutrient concentrations). Of these, division rate is a critical metric as it allows separation of the contributions of cell division and cell loss to changes in cell abundance. Division rate also reflects the physiological state of the cells; a low division rate indicates physiological limitation, and the relationships between division rate and environmental variables allow us to make inferences about which factors restrict growth at different times of year. We also analyze patterns of cellular PE fluorescence and volume, which provide additional insight into cellular physiology.

To guide our interpretation, we separate the annual cycle of abundance into four distinct seasons: spring bloom, summer cycles, fall fade, and winter wane. We choose these seasons based on patterns of cell abundance; spring is characterized by a dramatic increase, while summer, fall and winter decline in cell abundance, but with distinct features. In the following sections, we explore the dynamics within each season and find possible differences in the drivers of cell growth, physiology, and loss processes.

### Spring bloom

**Figure 6:**
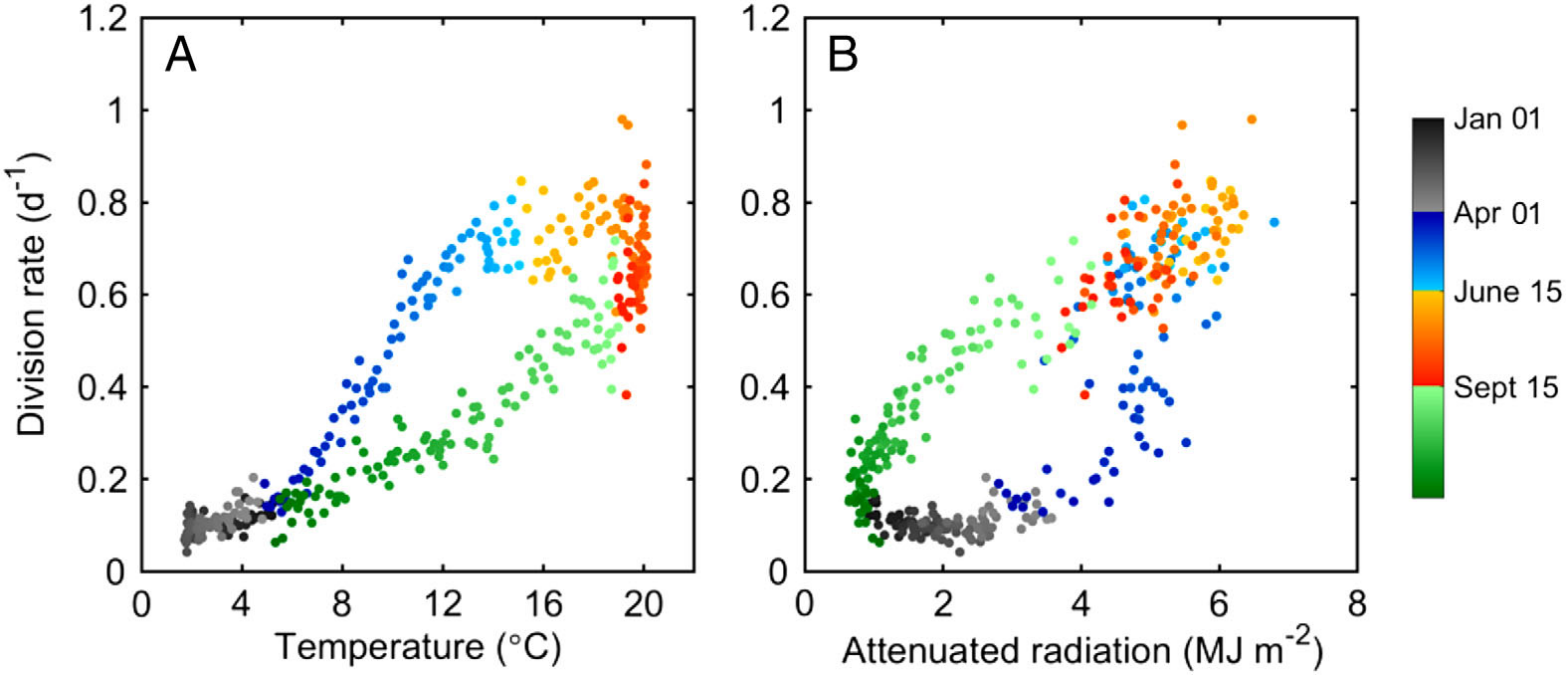
Relationships between daily climatologies. (**A**) *Synechococcus* division rate and temperature. (**B**) *Synechococcus* division rate and attenuated radiation from mean *K*_*d*_ values. Color denotes season and year day.

The spring bloom is characterized by a dramatic increase in cell concentration from a few hundred cells mL^−1^ in late March or early April up to ∼ 10^5^ cells mL^−1^ in June (Fig. 5A, Supporting Information Fig. S7). The underlying trigger for the bloom is the seasonal increase in water temperature (Hunter‐Cevera et al. 2016a). In particular, once water temperature reaches 5–6°C, division rate begins to increase (Fig. 6A). As described in Hunter‐Cevera et al. (2016a), we do not find any systematic relationship with radiation (Fig. 6B) or nutrients during this time, supporting temperature as the main driver of growth for the *Synechococcus* population in spring.

**Figure 7:**
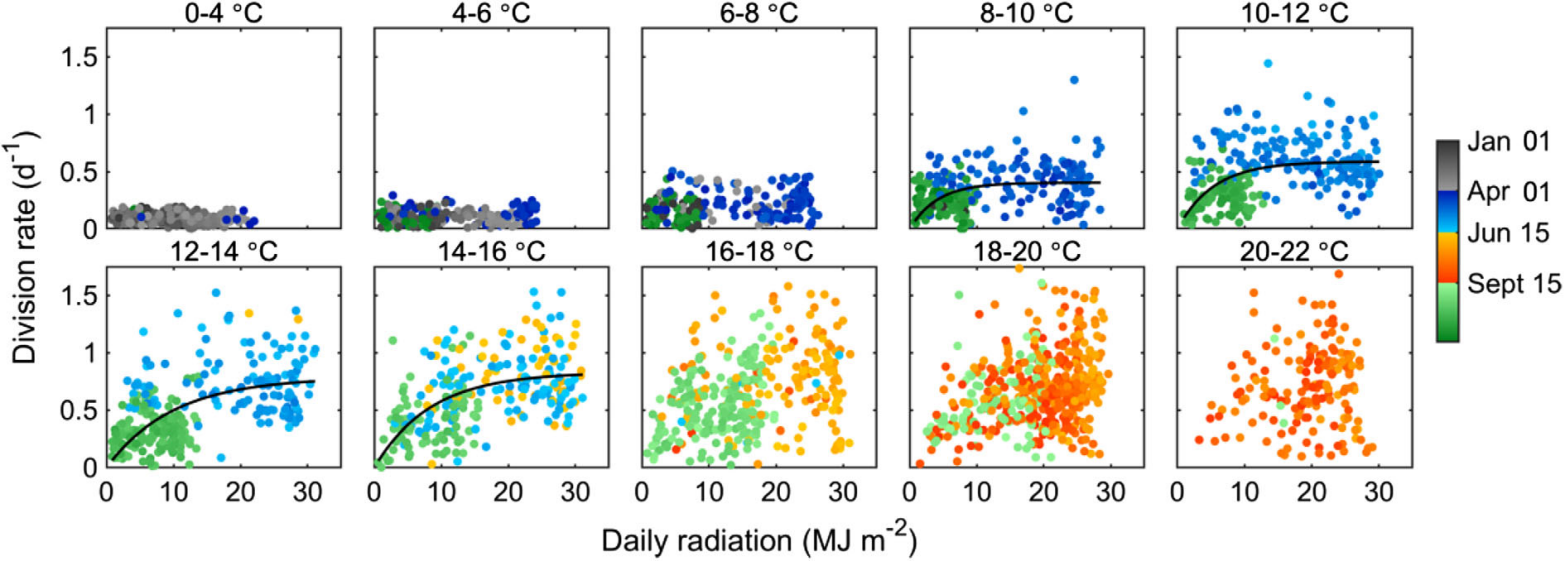
Relationships between daily division rate and incident solar radiation within 2°C water temperature intervals for daily points across all years. Color denotes season and year day. Black curves are minimized least square fits to Eq. 4 to guide interpretation, see “Materials and methods” section for details.

While light is not the main factor limiting *Synechococcus* division in spring, it can still have influence during this time. During the *Synechococcus* spring bloom in Woods Hole Harbor, Waterbury et al. (1986) observed a lower percentage of dividing cells during consecutive cloudy days, and observed disruption in the increase of *Synechococcus* cells following a major storm. This led Waterbury et al. to hypothesize that light could have short‐term effects on spring bloom trajectory. We also observe some spring days where light appears to be limiting. This is evident in the relationship between division rate and radiation for narrow temperature ranges (Fig. 7). Curves for ranges above a limiting growth temperature of 5–6°C resemble saturation responses (Fig. 7; e.g., 8–10°C, 10–12°C), with maximum values of division rate increasing with temperature (the plateau region of each curve in Fig. 7). In spring, division rate typically falls along what could be considered a “light saturated” portion of these curves. Occasionally during this season, we find points that fall along the “light limited” portion of the curves, where division rates are typically lower than might be expected for a given temperature. Fitted curves suggest this is most likely to occur when daily incident radiation is less than ∼ 10 MJ m^−2^ (roughly corresponding to a day with maximum irradiance of 500 W m^−2^, or equivalent to a clear sky day in late January).

**Figure 8:**
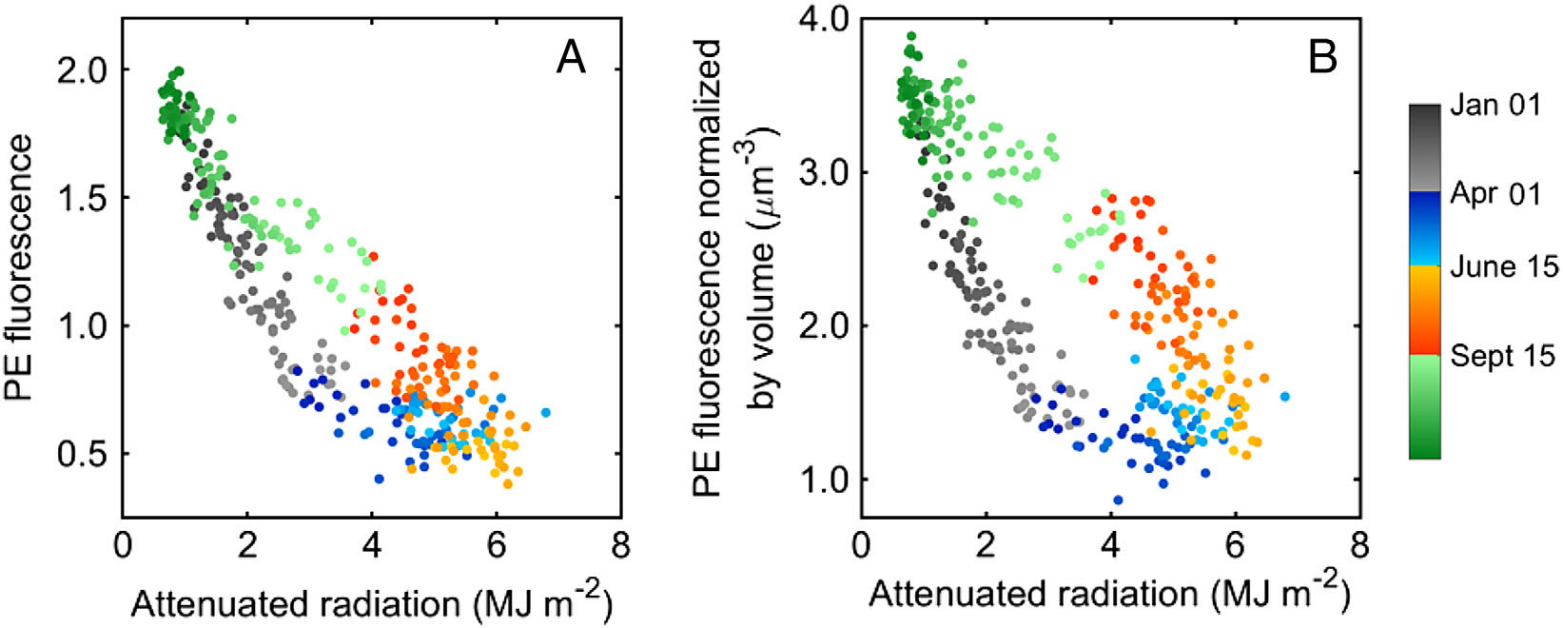
Relationship between climatology of attenuated radiation and (**A**) climatology of cellular PE fluorescence and (**B**) climatology of cellular PE fluorescence normalized to cell volume. Color denotes season and year day.

We find that cellular PE fluorescence (and PE fluorescence normalized to cell volume) typically reach minimum values during spring (Figs. 5F, 8). Fluorescence is a complicated phenomenon, influenced by pigment composition, efficiency of photosystems, nutrient stress, light acclimation, and other processes (Kirk 2011). With these caveats in mind, however, low PE fluorescence values would be consistent with cells that are light saturated, since to first order low fluorescence is linked to low intracellular pigment or reduced numbers of photosystems. This has been observed for light saturated *Synechococcus* cells in culture (Kana and Glibert 1987). More detailed measurements and (laboratory) manipulation would be needed though to further evaluate this hypothesis.

While the underlying trigger of the spring bloom is physiological, the accumulation of cells in this system is strongly influenced by loss processes. We calculate loss rates from the change in abundance and division rate, and therefore cannot partition between mortality from predation and the net effect of advection. We believe that the majority of losses though stem from ecological interactions rather than advection, as the latter would be unlikely to demonstrate such tight‐coupling with division rates (Hunter‐Cevera et al. 2016a). Loss rates begin to increase during spring, and this increase could be due to either heterotrophic grazers (e.g., nanoflagellates, ciliates, dinoflagellates) or viruses (Scanlan 2012), both of which have been shown to substantially contribute to *Synechococcus* mortality depending on time and location (Baudoux et al. 2008; Tsai et al. 2012, 2015; Pasulka et al. 2015; Mojica et al. 2016).

Loss rate closely tracks division rate in spring (Fig. 10B,E), but we notice an intriguing loss pattern. Loss rate is first able to match division rate when division rate begins to increase, but then rates diverge in late spring as the rate of increase in division rate outpaces loss rate. While this generalization is made from multiyear averages, we observe that many of the individual years in our data set undergo some variation of this pattern, where loss diverges only later after the initial increase in division rate (Supporting Information Figs. S11, S12). This divergence is small, average of 0.15 d^−1^, and allows for the accumulation of *Synechococcus* cells in the system. This close tracking is the basis for temporal shifts, rather than magnitude changes, of the spring bloom that we observe in our data set (Hunter‐Cevera et al. 2016a). We find a saturating pattern between loss rate and *Synechococcus* cell abundance once values exceed ∼ 10^3^ cells mL^−1^, suggesting that loss processes do not depend on concentration in the later half of the spring (Fig. 10G).

The initial and matched increase in loss rate could be due to increased grazer activity from increasing water temperatures (Rose and Caron 2007) and more active and abundant *Synechococcus* prey (Apple et al. 2011). Cyanophage could also be responsible for this pattern of loss rate in the form of emerging lysogens or resumption of infection from pseudolysogeny (Mann and Clokie 2012). At the start of spring, low cell abundances would be unlikely to support a purely lytic infection. Threshold concentrations of *Synechococcus* have been calculated to be 10^4^ cells mL^−1^ (Mann 2003), and studies of viral infections of natural populations are consistent with this estimate (Rodda 1996). McDaniel et al. 2002 found increased lysogeny of a *Synechococcus* population in Tampa Bay, Florida during cooler winter temperatures and lower host abundance. At MVCO, viruses facing unfavorable conditions in winter may also have incorporated as lysogens. Viral dynamics and life strategy strongly depend on host physiology (Mojica and Brussaard 2014), and changing temperatures or increased division rate may trigger induction of viruses.

We can further speculate about the reasons behind the deviation between division and loss rate later in spring. For grazers, availability of other prey, grazing attributes (ingestion curves, saturation thresholds) or increases in the predators of grazers, resulting in cascades that reduce grazing pressure on *Synechococcus*, could drive this deviation. Later in the spring, *Synechococcus* populations reach concentrations that in theory would be able to support lytic viral infections of susceptible hosts (Waterbury and Valois 1993). Accumulated net growth in late spring can vary from year to year in timing and duration, and likely indicate complicated dynamics between *Synechococcus* and their grazers or viruses (Supporting Information Fig. S11).

Cell abundance stops increasing once division rate has reached maximum values and loss rates are able to match in magnitude. This typically happens when water temperature reaches 15–17°C in mid‐June. For a *Synechococcus* isolate from MVCO, laboratory studies show that this strain can reach maximum division rates at ∼ 18°C (Hunter‐Cevera et al. 2014). This strain is believed to be representative of the type of *Synechococcus* that predominantly comprises the spring bloom (Hunter‐Cevera 2014). *Synechococcus* cell concentration typically does not increase above ∼ 3 × 10^5^ cells mL^−1^ at the end of the bloom. This may indicate a saturation concentration for which *Synechococcus* grazers are no longer food‐limited and can achieve their maximum ingestion rates. Maximum ingestion rates of nanoflagellates have been reported for prey concentrations ranges of 1–5 × 10^5^ cells mL^−1^ (Christaki et al. 2002; Tsai et al. 2008), which is at or just above the highest *Synechococcus* concentrations we observe at the end of the spring bloom at MVCO.

### Summer cycles

We define a summer season from mid‐June to mid‐September, when cell concentration remains relatively high (> 5 × 10^4^ cells mL^−1^), but still exhibits a general decline as fall approaches (Fig. 5A, Supporting Information Fig. S8). The abundance pattern during this time is also characterized by highly variable, short timescale fluctuations (∼ 2–3 week periods) that can result in an order of magnitude change in cell concentration (Supporting Information Fig. S8). Variations in either division rate or loss rate could produce these dynamics. For some years, we notice a dramatic decline in cell concentration following the height of the spring bloom (e.g., 2003, 2012, 2017). In other years, we find that cell concentration values at the end of the bloom are able to persist for longer periods into summer (e.g., 2005, 2011, 2018).

In summer, the abundance of *Synechococcus* cells enables them to substantially contribute to primary production in this area. A rough estimate for *Synechococcus* primary production can be obtained by multiplying the number of new cells produced per day by cell volume by a carbon:volume ratio (*see* Supporting Information for more details). This approach yields conservative estimates of ∼ 5–10 mg C m^−3^ d^−1^. Compared with regional estimates obtained from the MARMAP program (O'Reilly et al. 1987; O'Malley 2017), in summer, *Synechococcus* could comprise between 3% and 25% of total primary production in this area. These percentages are consistent with what others have reported for *Synechococcus* contributions in coastal systems (Li 1994; Jardillier et al. 2010). From our estimates, we would expect *Synechococcus* to be responsible for a sizeable fraction of primary production while cell concentration is greater than ∼ 5 × 10^4^ cells mL^−1^.

Division rates are generally high (0.7–1 d^−1^) in this season, but can also be quite variable and somewhat lower than expected for a given temperature and light (Fig. 7). This suggests that other environmental variables, possibly nutrients, can affect division rate in the summer. Low division rates can occur interspersed among moderate or high rates in the summer, and occasionally for multiple days in a row.

Variations of loss rate occur at the same frequency as division rate, and could contribute to observed fluctuations in cell concentration in summer. As in spring, changes in loss rate could result from grazing saturation thresholds, decreases or increases in concentration of grazers, activity of viruses or patchiness, and advection of water. The fluctuations in concentration can be somewhat regular, with peaks in abundance separated on average by 2 weeks. Predator‐prey dynamics can produce such oscillations (Kot 2001). Viral infection could also generate these patterns, as above 10^4^ cells mL^−1^, susceptible populations should experience significant losses from phage (Mann 2003). Many viruses are thought to be specific to certain *Synechococcus* types (Mühling et al. 2005). With a particularly diverse *Synechococcus* population at MVCO in summer (Hunter‐Cevera et al. 2016b), these periodic decreases could be a result of viral lysis of certain types of *Synechococcus* or attacks on sensitive types that have temporarily been allowed to increase (Waterbury and Valois 1993).

The spareness of our nutrient data makes it difficult to evaluate if low division rates result from resource limitation. If nutrient limitation does occur, it does not appear to be systematic. We find further support of consistently nutrient‐replete cells from values of cellular PE fluorescence in summer. Beginning around July and continuing into the fall, PE fluorescence values on average begin to increase (Figs. 5, 8). To the extent these fluorescence values reflect intracellular pigment quota, this increase in fluorescence would indicate sufficient nutrients to keep and build required pigment and photosynthetic machinery. Studies investigating nutrient limitation of natural phytoplankton populations often find an increase in PE and chlorophyll fluorescence for *Synechococcus* upon nutrient enrichment (Graziano et al. 1996; Bonnet et al. 2008; Davey et al. 2008), such that higher fluorescence values here may indicate replete cells.

We note that cellular PE fluorescence values in late summer are generally higher than for the same light levels encountered in early spring (Fig. 8A). One explanation may involve temperature and cellular demand for photosynthate. In spring, growth is temperature‐limited, and extra pigment could lead to unnecessary and potentially damaging absorption of excess light energy. In summer, cells should not be limited by temperature (as evidenced by higher division rates, Fig. 6A). A higher rate of cell division would require more photosynthate, possibly requiring changes to the photosynthetic machinery (i.e., increased pigment production, increased number of photosystems) to supply this demand. Our observations of higher fluorescence values per volume may reflect such changes (Fig. 8B).

**Figure 9:**
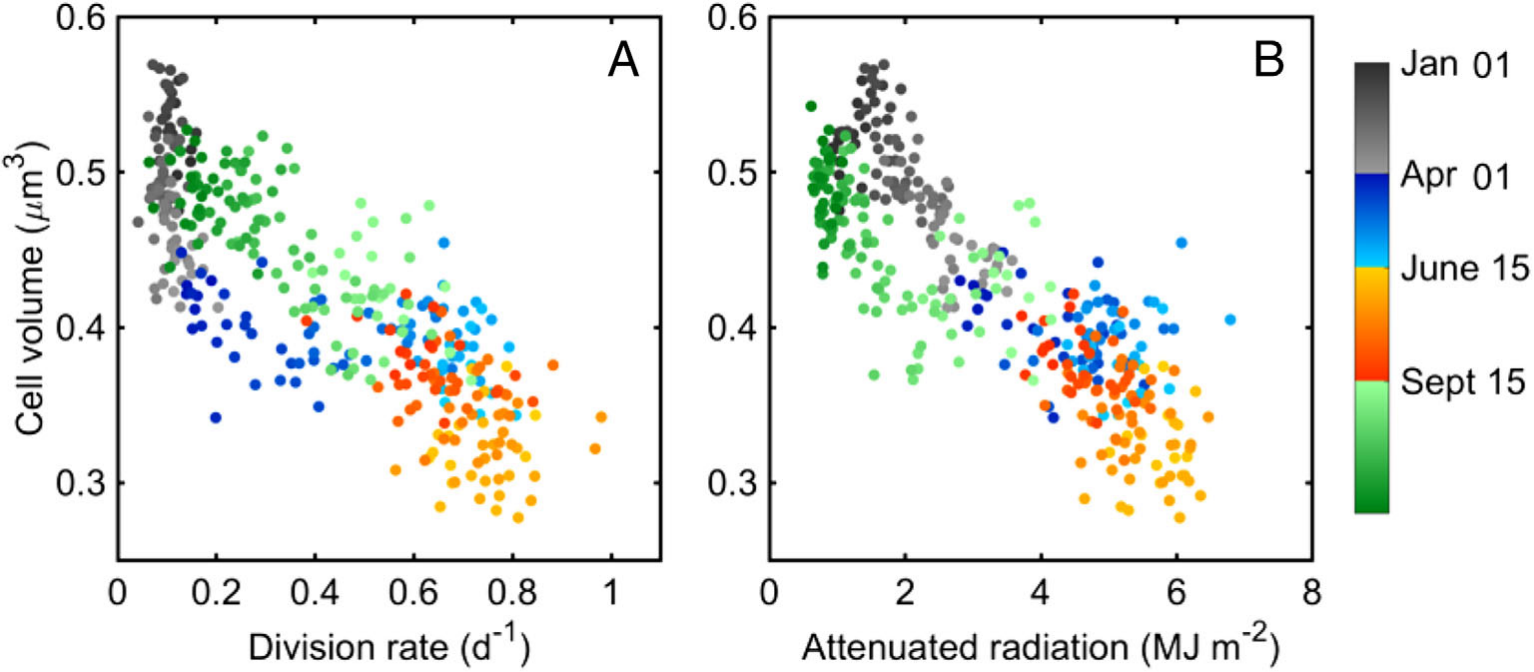
Relationship between climatologies of cell volume and (**A**) daily division rate and (**B**) attenuated radiation. Color denotes season and year day.

*Synechococcus* at MVCO undergo a repeated annual pattern in cell volume, in addition to the daily changes in cell volume that result from growth and division. The variation observed within this annual pattern is generally larger (although not always) than the diel change in volume (Supporting Information Fig. S13). We find that *Synechococcus* cell volume is generally the smallest during the summer (Fig. 5E). Cell volume is intrinsically tied to division rate; cells must accumulate resources before cell division can occur. Broadly across phytoplankton taxa, smaller cells tend to have higher division rates, which is thought to result from metabolic scaling (Finkel et al. 2010), although there are many exceptions in the phytoplankton (Chisholm 1992). While we find a general negative relationship between division rate and cell volume for *Synechococcus*, we note that this relationship does not necessarily hold within individual seasons (Fig. 9A). Division rate does not appear to relate to cell volume in winter, spring, or summer, nor does it show a relationship with temperature (Supporting Information Fig. S14A). We do find a relationship, however, between cell volume and radiation; cell volume decreases with increasing radiation for every season except spring (Fig. 9B). This suggests that *Synechococcus* could be regulating their volume in response to light environment. In field studies examining cells with FCM, *Synechococcus* cells at the base of the euphotic zone had larger forward scattering (representative of volume) than cells at the surface (Olson et al. 1990b). In culture, *Synechococcus* cells increased in volume under decreasing, growth‐limiting irradiance (Kana and Glibert 1987; Morel et al. 1993). For strain WH7803, this increase in cell volume was accompanied by increase in thylakoid bands within the cell (Kana and Glibert 1987). Freshwater cyanobacteria also adjust cell volume and shape in response to light intensity; *Fremyella diplosiphon* cells elongate in low light and become spherical in high light (Montgomery 2015). These changes are thought to regulate photosynthetic capacity by either increasing or reducing membrane availability (Montgomery 2015).

Another explanation for the patterns in cellular PE fluorescence and cell volume could be changes in the composition of the *Synechococcus* assemblage itself. *Synechococcus* is a diverse genus, and at MVCO, 13 different clades have been identified from either environmental clone libraries or culture isolations (Hunter‐Cevera et al. 2016b). Clades can differ in various aspects of their physiology, such as temperature growth response, nutrient acquisition, and photoacclimation or wavelength preferences (Palenik 2001; Moore et al. 2002; Pittera et al. 2014). Characterization of the bacterial community at MVCO (from high throughput sequencing of the V6 region within the 16S rRNA gene) over three seasonal cycles indicated that the diversity of the *Synechococcus* population changes over the year, and diversity was highest in late summer (Hunter‐Cevera 2014). Further study is needed to determine if the clades present in summer at MVCO have different pigmentation, associated fluorescence characteristics and intrinsic cell volume than those found in spring. Investigations using multiple lasers and other filter sets (such as in Olson et al. 1988) could provide this information. We note that shifts in diversity may be detected within our model. For many days, we find that one population appears sufficient to reproduce diel volume patterns (Supporting Information Fig. S17), but in summer, starting proportions of subpopulations tend to be more equal. During this season, two components appear necessary to capture volume dynamics, which could reflect different clade ecophysiologies. We stress though that each subpopulation in our model is likely a composite of several clades and further validation must be done to interpret subpopulation model parameters and division rates.

### Fall fade

Through late September into January, cell concentration can be similar in magnitude to summertime, but values are typically lower (∼ 10^4^ cells mL^−1^) and exhibit a slow decline over the season (Fig. 5A, Supporting Information Fig. S9). The decline in cell concentration can be attributed to a systematic decline in division rates that begins in October. Division rates show a strong correlation with both temperature and light (Fig. 6A,B), but compared to the same temperature encountered in spring, division rates are lower in fall. This suggests that light is a limiting factor for division rate during this season. From the relationship between division rate and incident radiation for narrow temperature windows (Fig. 7), we find that the majority of division rates during fall are along the “light limited” portion of these curves (in contrast to spring when points are typically on the saturated portion).

Values of cellular PE fluorescence also support light limitation in fall. In this season, PE fluorescence continues to increase until it reaches maximum values in December, corresponding with decreasing light levels (Fig. 8; we find no relationship with PE fluorescence and temperature; Supporting Information Fig. S14B). As mentioned above, fluorescence is determined by many factors (nutrient status of cell, previous radiation conditions, temperature, pigment composition), but to a first approximation, this pattern suggests *Synechococcus* cells are maximizing their ability to capture light during this time. We also note a general increase in cell volume during the fall, which could be associated with a membrane increase for additional photosynthetic capacity. In the fall, cells would not only be responding to the seasonal decline in incident radiation but also to further reduction in light penetration through the water column as attenuation coefficients increase (Fig. 2). We believe that the seasonal increase in attenuation is mainly due to an increase in chlorophyll from the growth of larger, eukaryotic phytoplankton during this time (Sosik unpubl.).

The combined effects of decreasing temperature and declining light make interpretation of interannual variation (and future prediction) for this season difficult. For example, anomalies of daily temperature and *Synechococcus* division rate and abundance only demonstrate weak relationships during this time, despite temperature being a controlling factor (Supporting Information Fig. S15). Climatological relationships between *Synechococcus* properties and incident radiation also differ slightly from those calculated with attenuated radiation (Supporting Information Fig. S16). We therefore cannot readily disentangle the effect of light without knowledge of the in situ light environment (including mixing environment), highlighting the need for real‐time monitoring of these variables.

**Figure 10:**
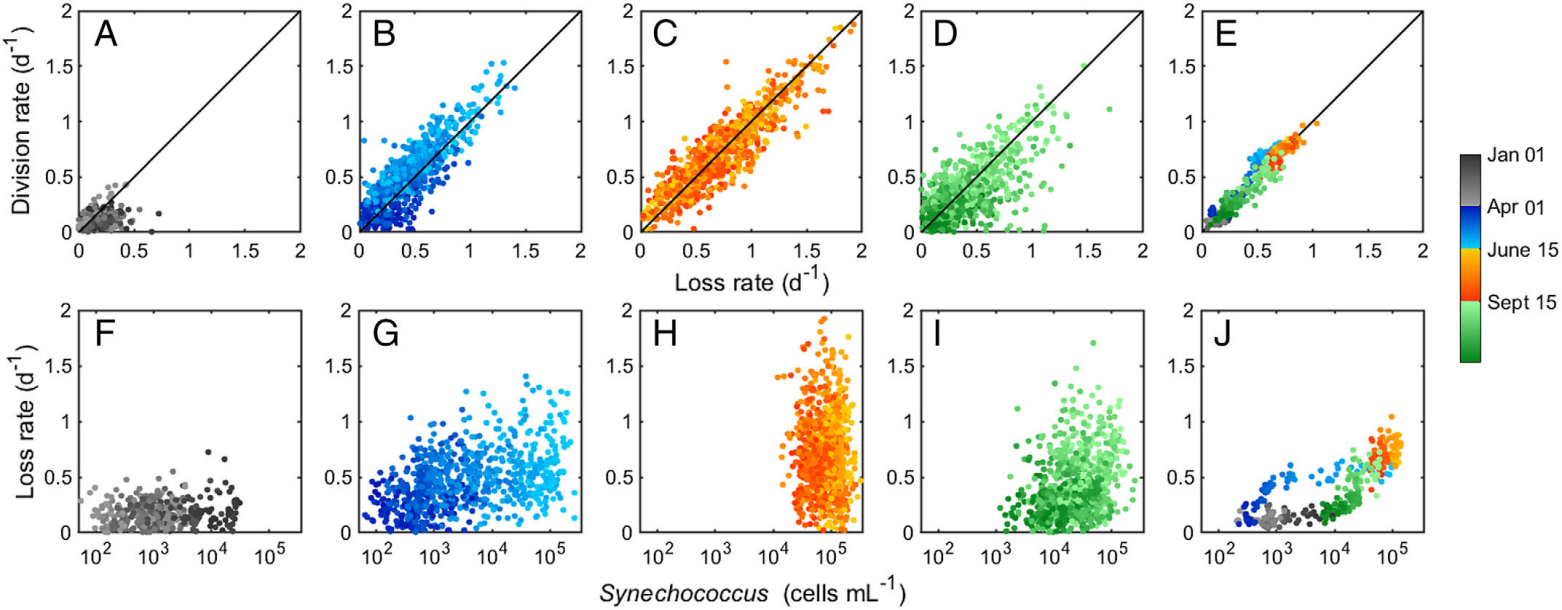
Top panels (**A**–**E**) are relationships between division and loss rates by season. Black line is one‐to‐one line for reference. Bottom panels (**F**–**J**) are relationships between loss rate and *Synechococcus* cell concentration by season. Daily points are plotted for winter (**A**, **F**), spring (**B**, **G**), summer (**C**, **H**), and fall (**D**, **I**). Climatological relationships are shown for division and loss rate (**E**) and loss rate and cell concentration (**J**). Color indicates season and year day.

Loss rates follow division rate in magnitude, but loss rates are slighter higher, resulting in the slow decline of cell concentration (Fig. 10D). It remains an open question why loss rates decrease during this season and are able to match division rates so closely. Loss due to grazing depends on the concentration and activity of grazers and prey availability. While *Synechococcus* concentrations in fall are still within the range for relatively high ingestion rates by micrograzers (Christaki et al. 2002; Tsai et al. 2008), the average relationship between loss rate and *Synechococcus* cell concentration displays a linear relationship (Fig. 10J), indicating that grazing may be limited by prey availability. While this is not an ingestion rate curve, it lends support to loss processes being affected by abundance of *Synechococcus* at this time of year. We also note the difference of this relationship between fall and spring, which may reflect different sources of mortality within seasons (i.e., loss to due to grazers or viral infection, grazers with different ingestion capabilities or prey preferences, etc.).

### Winter wane

Compared to the fall, we observe a steeper decline in cell concentration beginning in January. Minimum cell concentrations (100–1000 cells mL^−1^) are reached in March or beginning of April (Fig. 5A, Supporting Information Fig. S10). This decline is due to very low division rates (0.05–0.15 d^−1^) coupled to loss rates that are just higher than division rate (0.15–0.2 d^−1^). No relationship is apparent between division rate and light during winter (Figs. 6, 7), consistent with temperature limiting growth at this time.

**Figure 11:**
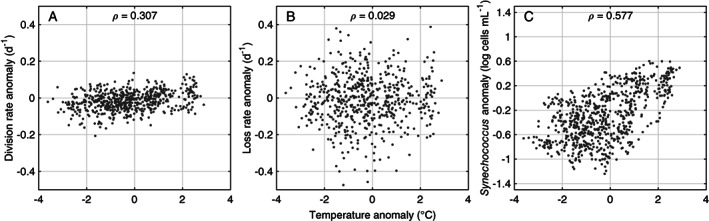
Relationship between daily *Synechococcus* anomalies and daily temperature anomalies for time periods with temperature below 6°C. (**A**) Division rate anomaly. (**B**) Loss rate anomaly. (**c**) *Synechococcus* concentration anomaly. Values for Pearson's correlation coefficient (*ρ*) between anomalies are presented at the top of each plot.

For the years we observed, we find one of two general patterns each winter: either cell concentration is able to stay relatively high, at ∼ 1000 cells mL^−1^ or it continues to drop to a few hundred cells mL^−1^. Anomalies in cell abundance during winter are correlated with temperature anomalies (as are division rate anomalies) (Fig. 11). This suggests that the minimum cell concentration reached is primarily determined by how cold the winter is. As the trigger temperature of the spring bloom appears to be ∼ 5°C, warmer winters that hover at 4–5°C may allow *Synechococcus* cells to slowly replace themselves, leading to relatively high winter cell concentrations. Paulsen et al. (2016) found slightly positive net growth for *Synechococcus* in Arctic waters (0–2°C), indicating viable cells even below 5°C. To our knowledge, no studies have systematically investigated how marine *Synechococcus* grow or survive at very cold temperatures (0–5°C). Investigation of such physiology would contribute to our understanding of how dormancy or tolerance impacts cell abundance as well as the geographic range of *Synechococcus*.

Cellular PE fluorescence decreases during winter from close to maximum values in January to minimum values in April (Fig. 8A). We also find that PE fluorescence values are lower in winter than in fall for the same light level, especially when values are normalized to cell volume (Fig. 8B). This reduction in fluorescence could be indicative of a stress response. Cold temperatures pose a challenge for photosynthetic organisms as incoming light energy must be balanced with a reduced cellular demand. As division rates are low in winter, this energy must be dissipated in order to avoid cellular damage. Lower pigment amounts or changes in number of photosystems may prevent excess energy from entering the system, a common strategy among phytoplankton to cold acclimation (Morgan‐Kiss et al. 2006). Pittera et al. (2014) found that *Synechococcus* cells subjected to a temperature drop from 22°C to 13°C demonstrated a wide range of photoacclimation strategies to accommodate reduced cellular demand, reflected by observations of reduced red and orange cellular fluorescence. We also observe decreases in cell volume throughout the winter, which could be associated with membrane reduction to reduce light absorption.

Loss rates are low in winter, but also highly variable. Grazers could still be active, as psychrophilic nanoflagellates have been observed (Choi and Peters 1992), but *Synechococcus* concentrations are very low, such that grazers targeting *Synechococcus* are likely food‐limited. Tsai et al. (2008) found that in cooler water (∼ 16°C) off the coast of Taiwan, when *Synechococcus* division rate was low for this system, bacteria made up a larger portion of prey items for heterotrophic nanoflagellates than *Synechococcus*. Lytic viruses would also have difficulty sustaining infection under these low *Synechococcus* concentrations (Mann 2003), although emerging lysogens throughout the winter is a potential source of loss. Advection and other physical processes may also serve as a source of loss or dilution of the *Synechococcus* population with water masses containing lower *Synechococcus* concentrations.

## *Conclusions*

The strikingly regular pattern of *Synechococcus* cell concentration changes on the New England continental shelf can be attributed to different seasonal controls on growth coupled to the timing and balance of loss processes. Our results are consistent with temperature being the main factor determining growth in the winter and spring, with light becoming limiting in the fall. We also find seasonality in losses to the *Synechococcus* population; a temporal offset from increasing division rates enables *Synechococcus* cells to accumulate and bloom in the spring, while tightly coupled losses to declining division rates in fall result in a slow decline of cell abundance.

The importance of loss to population dynamics highlights the need to examine the identity, abundance, and activity of *Synechococcus* predators. Answers to these questions will not only provide insight into the mechanisms behind observed loss patterns, but also understanding of how carbon fixed by *Synechococcus* is transferred within coastal food webs. Heterotrophic grazers enable more direct transfer to higher trophic levels, while viruses divert it to the microbial loop and subsequent heterotrophic bacteria uptake.

Knowledge of abundance dynamics identifies various selection pressures on *Synechococcus* populations, which may help us understand why this genus, along with *Prochlorococcus*, harbors incredible diversity in physiological and ecological traits. The population at MVCO is comprised multiple, distinct clades, each of which is thought to have different ecophysiologies and preferred environmental niches. The role of such microdiversity in this group (and other marine microbes) is still an open question in microbial ecology. Insight into growth limitations, predation, and how both change over the annual cycle will help us better understand why different ecotypes exist and how they contribute to an overall robust population.

**Figure 12:**
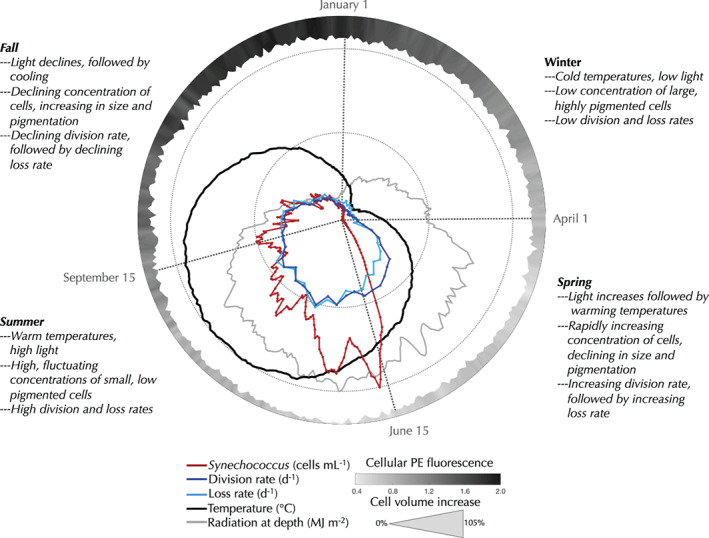
Circular representation of *Synechococcus* properties and environmental conditions at MVCO; daily climatology of *Synechococcus* cell concentration, linear scale (red line), weekly climatologies of division rate (dark blue line) and loss rate (light blue line), daily temperature climatology (black line), and running mean (5‐d window) of estimated daily radiation at depth (light gray line). Daily climatology of cellular volume is indicated by width of outer gray‐shaded circle relative to minimum value of 0.278 *μ*m^3^. Shading indicates climatological values of cellular PE fluorescence, see color bar. Scaling is arbitrary and relative to each variable. For reference, inner dotted circle indicates a division or loss rate of 0.82 d^−1^, and outer dotted circle indicates maximum climatological values for *Synechococcus* concentration (1.4 × 10^4^ cells mL^−1^), temperature (20.12°C), and light at depth (6.15 MJ m^−2^).

While open questions remain about key ecological aspects for the *Synechococcus* population at MVCO, we have provided a testable working model and framework (summarized in Fig. 12) to understand the annual cycle of cell abundance in temperate regions. This enables interpretation of longer (annual) and shorter (weeks–months) abundance changes in the context of underlying seasonal controls. This framework will be useful for model development to predict picocyanobacteria abundances and how they may change in the future. Further understanding into the abiotic and biotic factors that affect microbial dynamics will require development of technology that allows for in situ environmental investigation. Automation combined with existing technologies offers exciting opportunities to observe a broad range of cell characteristics and environments at the space and timescales that matter for these organisms (Breier et al. 2014; McQuillan and Robidart 2017). Continued development of such technology will enable better synergy between observations made at small scales and our understanding of large‐scale ecosystem functioning and processing.

## Conflict of Interest

None declared.

## Supporting information


**Appendix** S1: Supporting Information
