# Supplementary material for: Seasons of Syn
Source: Limnol Oceanogr. 2019 Nov 19;65(5):1085–102. doi: 10.1002/lno.11374 (PMC7319482; doi:10.1002/lno.11374)
Supplement: Supplementary file 1 — Appendix S1: Supporting Information [file LNO-65-1085-s001.pdf]

Supplementary Information

Primary production estimate

For a 'back-of-the-envelope' estimation of daily *Synechococcus* primary production, we can multiply daily division rate, cell concentration at dawn, cell volume at dawn and a carbon:volume ratio. The rationale is as follows: a cell at dawn is likely to just have divided during the previous night and will accumulate carbon biomass during the day. On day t, for a daily division rate μ, and a starting concentration of $N(t)$ cells, $N(t)\exp(\mu \cdot t) - N(t)$ new cells would be produced in the day. Assuming that these new cells had to be somewhat equal in volume to the original size of the mother cell at dawn, we can multiply the following constructs together to arrive at a daily primary production estimate:

$$\bar{\mathrm{P}}\mathrm{P}(t) = C \cdot V(t_{\mathrm{dawn}}) \cdot N(t_{\mathrm{dawn}})(\exp(\mu \cdot t) - 1) \tag{5}$$
$$\frac{\mathrm{mg\ C}}{\mathrm{m}^3\ \mathrm{d}} = \frac{\mathrm{C\ fg}}{\mu\mathrm{m}^3} \cdot \frac{10^{-12}\ \mathrm{mg}}{\mathrm{fg}} \cdot \frac{\mu\mathrm{m}^3}{\mathrm{cell}} \cdot \frac{\mathrm{cells}}{\mathrm{mL}} \cdot \frac{10^6\ \mathrm{mL}}{\mathrm{m}^3} \text{ per day,}$$

where C is a carbon:volume estimate for *Synechococcus*, $V(t_{\mathrm{dawn}})$ is the mode of cell volume at dawn, and $N(t_{\mathrm{dawn}})$ is the concentration of *Synechococcccus* at dawn. Eqn. 5 displays units for ease of conversion. This estimate would not include cells that are fixing carbon but did not divide during the day.

We use the carbon:volume estimates from Heldal et al. 2003 of 150-290 fg C μm^{-3} (from strains WH7803, WH8103). With a mid-range value of 220 fg C μm^{-3}, carbon per *Synechococcus* cell would be $\sim 60 - 160$ fg C cell^{-1} over an annual cycle. These values would be on the lower end of reported values for carbon content per cell of cultured *Synechococcus* of 77-250 fg C cell^{-1} (Bertilsson et al. 2003; Fu et al. 2007; Lopez et al. 2016), and may be do to inherent differences between the *Synechococcus* strains found at MVCO and those used in these studies as well as differences between cultured and wild cells.

From the mid-range value of 220 fg C μm^{-3}, we estimate *Synechococcus* primary production to be $\sim$ 5-10 mg C m^{-3} d^{-1} in the summer, although estimates are highly variable (see Table S1). We compared these estimates of *Synechococcus* primary production with estimates of total primary production obtained from C-14 incubations during cruises of the Marine Resources Monitoring, Assessment, and Prediction (MARMAP) program of the NOAA Northeast Fisheries Science Center, undertaken from 1977-1982 (O'Reilly et al. 1987; O'Malley 2017). We use surface estimates (< 5 m) that are within $\sim 1°$ latitude and longitude of MVCO and would be representative of surrounding water (specifically those found between 41 - 41.5°N and 70.25 - 71.5°W). Estimates were also highly variable across years, with lowest mean values found in June of $\sim$34 mg C m^{-3} d^{-1} and highest in August of $\sim$219 mg C m^{-3} d^{-1} for this area. *Synechococcus* primary production could roughly be between 3-25% of total primary production, although both datasets have substantial variation, making bulk monthly comparisons difficult.

Table S1: Monthly mean and standard deviations of primary production (PP) estimates for area total (MARMAP dataset) and *Synehcococcus* at MVCO, calculated from Eqn. 5.

Month	PP MARMAP (mg C m^{-3} d^{-1})	PP *Syn* (mg C m^{-3} d^{-1})
June	34.3 ± 22.2	8.8 ± 9.1
July	82.8 ± 49.5	9.4 ± 7.0
August	219 ± 34.9	6.5 ± 4.8
September	45.9 ± 18.9	4.5 ± 3.9

Figures

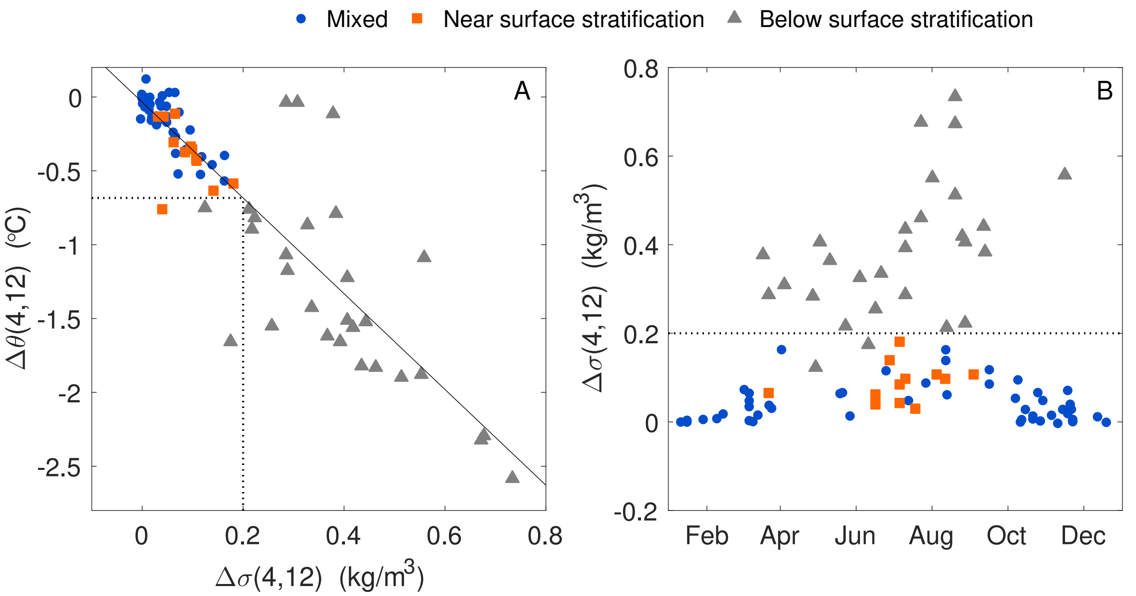

Figure S1: A) Relationship between temperature difference ($\Delta\theta$) and potential density difference ($\Delta\sigma$) at 4 m and 12 m from available CTD casts. Solid line is linear regression fit. Dashed lines emphasize that $\Delta\sigma = 0.2$ kg/m^3 corresponds to $\Delta\theta = 0.68$ °C. B) Potential density differences between 4 m and 12 m, plotted by yearday. Dashed line indicates threshold value of 0.2 kg/m^3 for $\Delta\sigma$. Symbol color and shape indicates density profile classification as either mixed (blue circles), near surface stratified (orange squares) or stratified at depth (gray triangles).

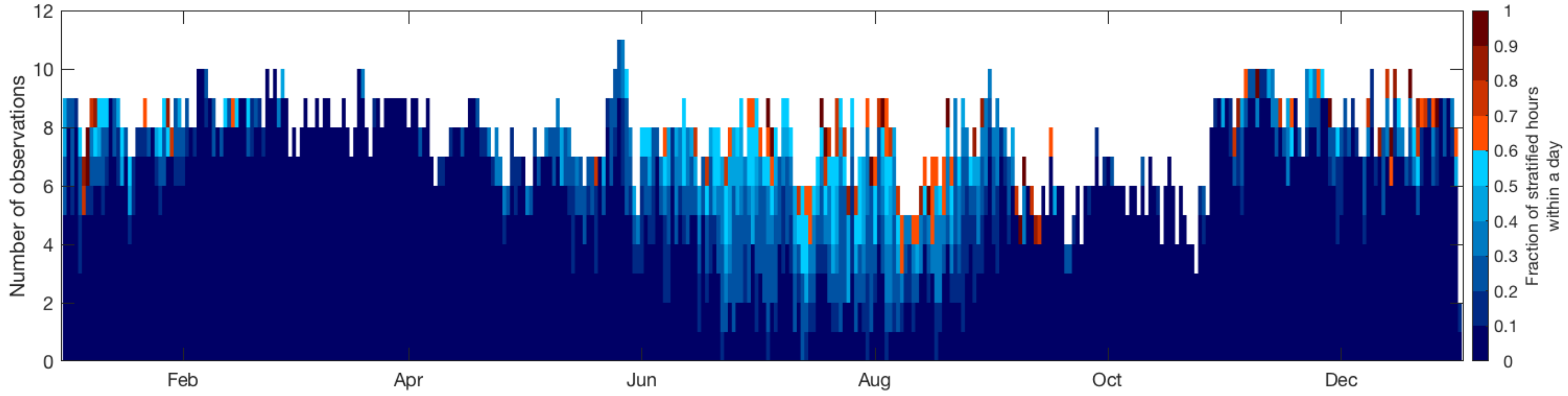

Figure S2: Distribution of year days within time series for which observations are available. Days are classified as stratified (orange shaded bars) or not stratified (blue shaded bars). Shade of color indicates the fraction of daylight hours for which hourly temperature difference between 4 and 12 m was > 0.68°C.

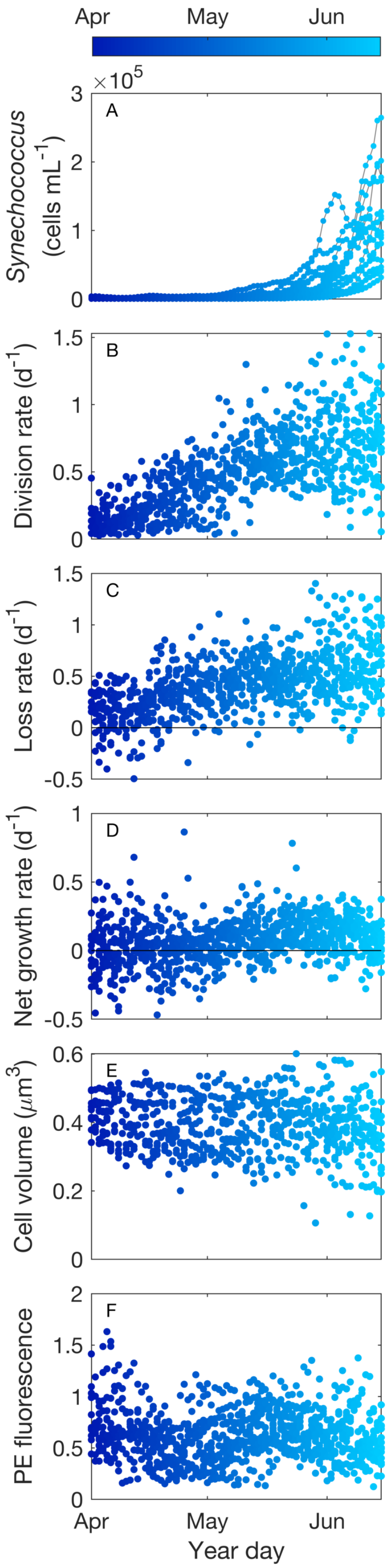

Figure S3: Scatter plots of A) *Synechococcus* concentration B) division rate C) loss rate D) net growth rate E) cell volume and F) cellular PE fluorescence by year day for data from 2003-2018 for spring.

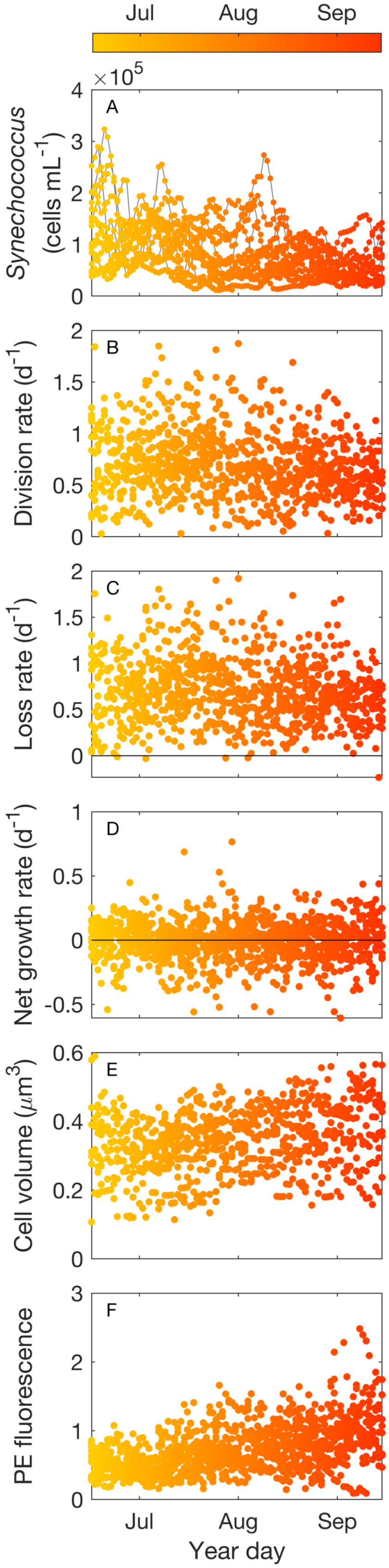

Figure S4: Scatter plots of A) *Synechococcus* concentration B) division rate C) loss rate D) net growth rate E) cell volume and F) cellular PE fluorescence by year day for data from 2003-2018 for summer.

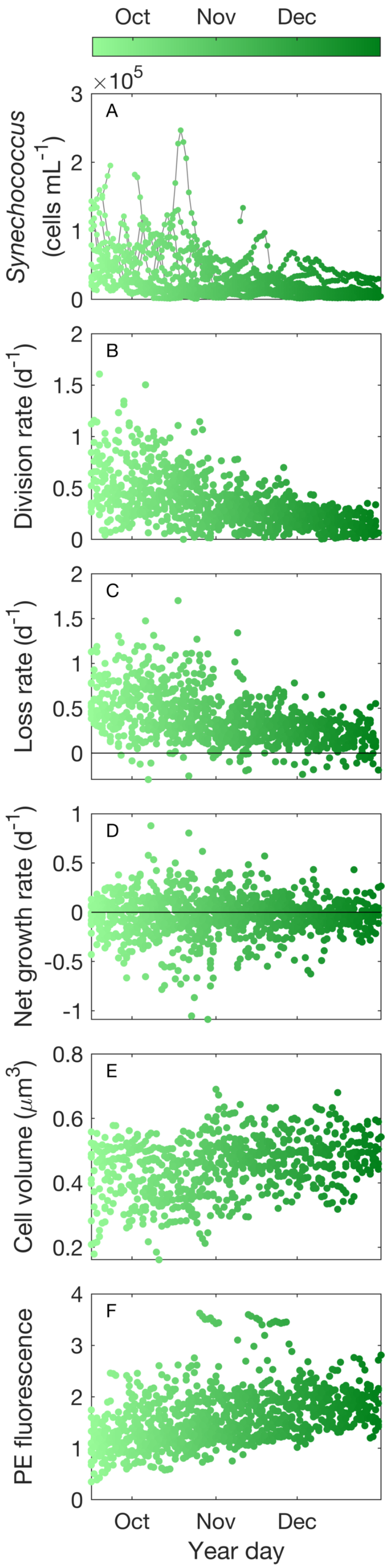

Figure S5: Scatter plots of A) *Synechococcus* concentration B) division rate C) loss rate D) net growth rate E) cell volume and F) cellular PE fluorescence by year day for data from 2003-2018 for fall.

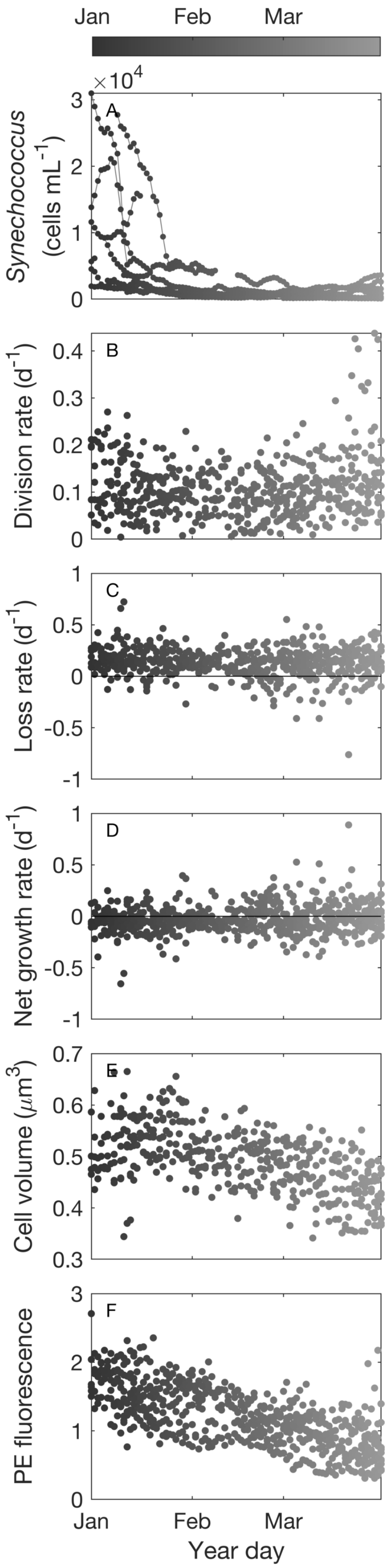

Figure S6: Scatter plots of A) *Synechococcus* concentration B) division rate C) loss rate D) net growth rate E) cell volume and F) cellular PE fluorescence by year day for data from 2003-2018 for winter.

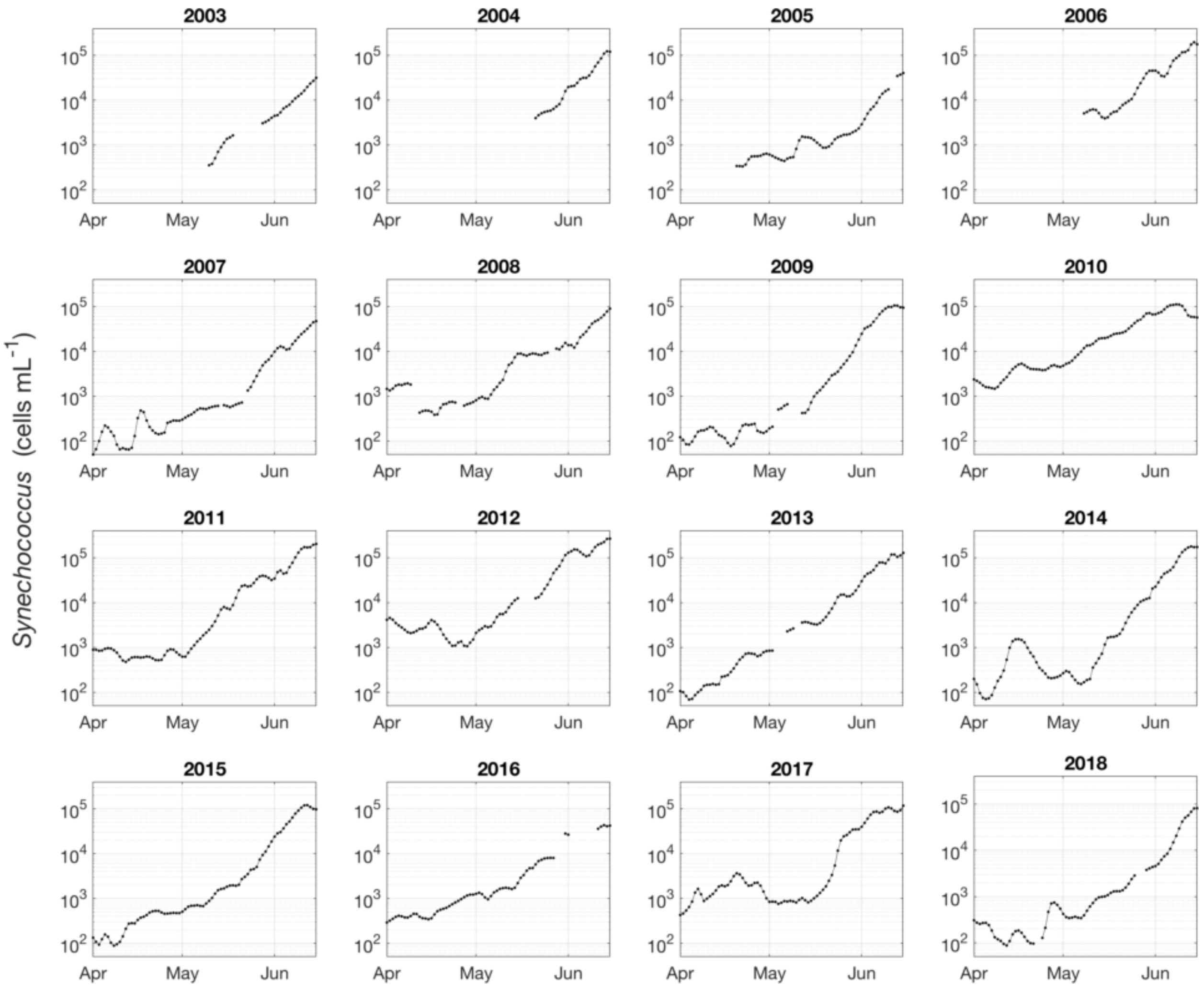

Figure S7: *Synechococcus* cell concentration during spring months for each year in dataset, log scale.

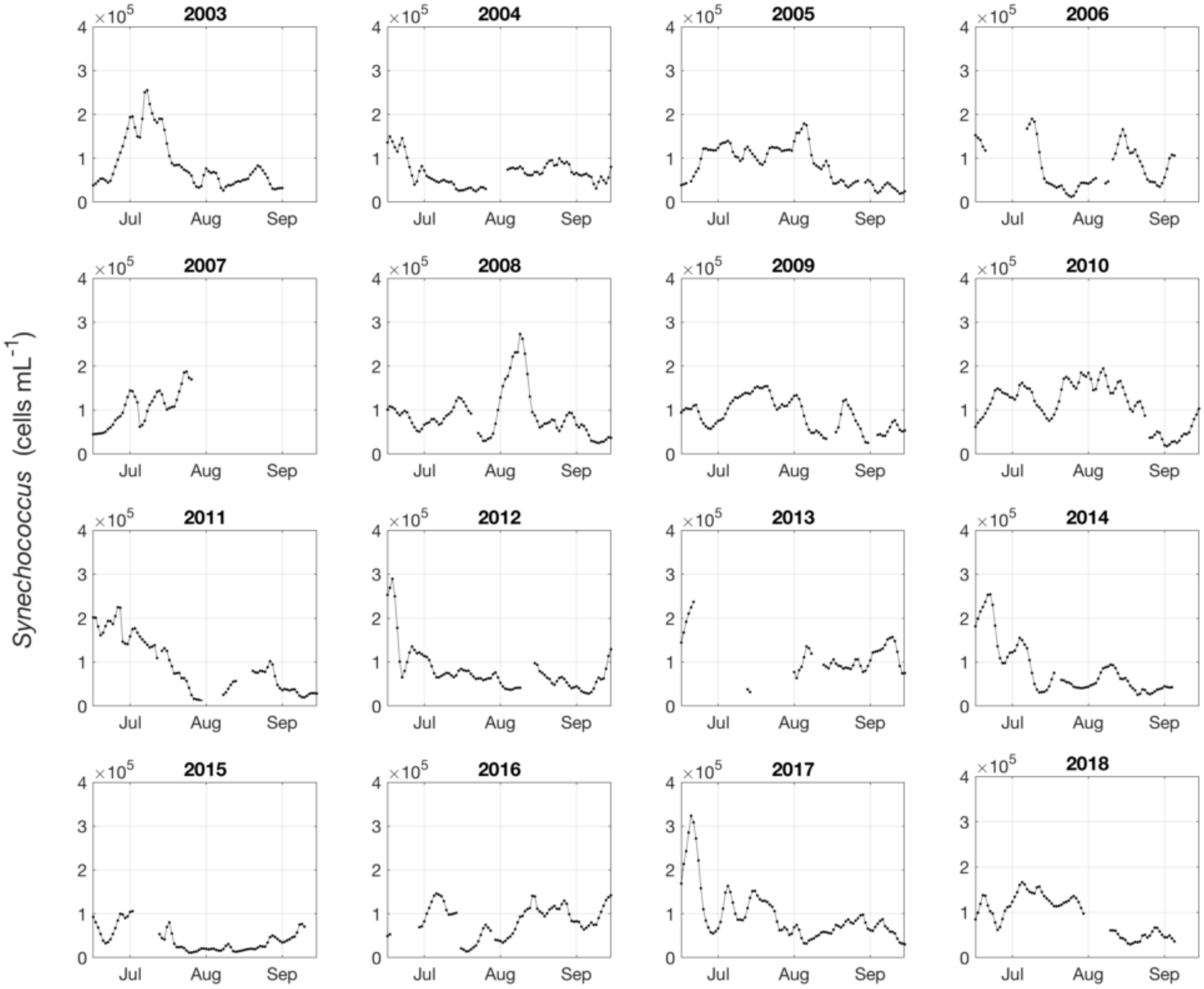

Figure S8: *Synechococcus* cell concentration during summer months for each year in dataset, linear scale.

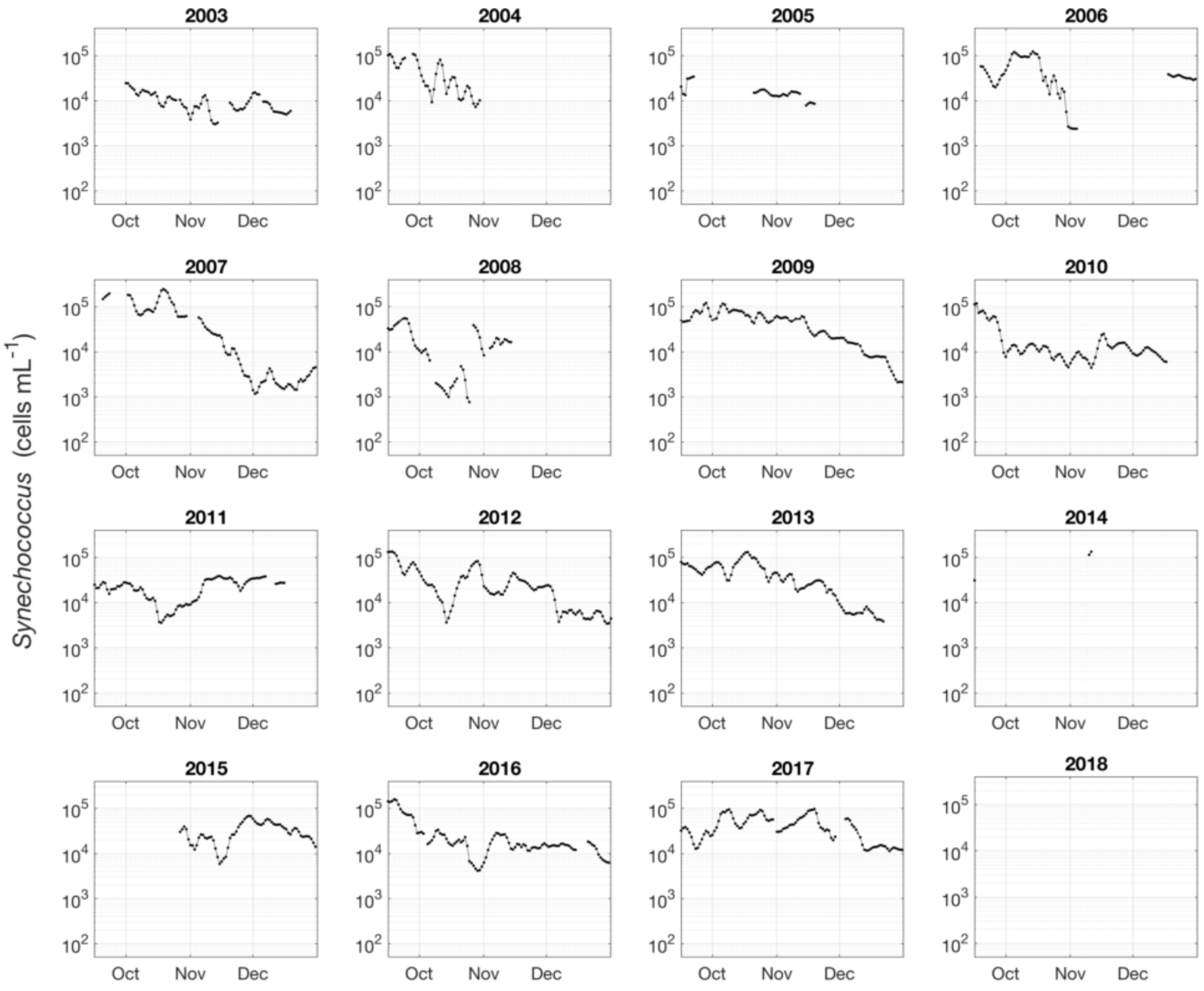

Figure S9: *Synechococcus* cell concentration during fall months for each year in dataset, log scale. Note limited data for 2014, 2018.

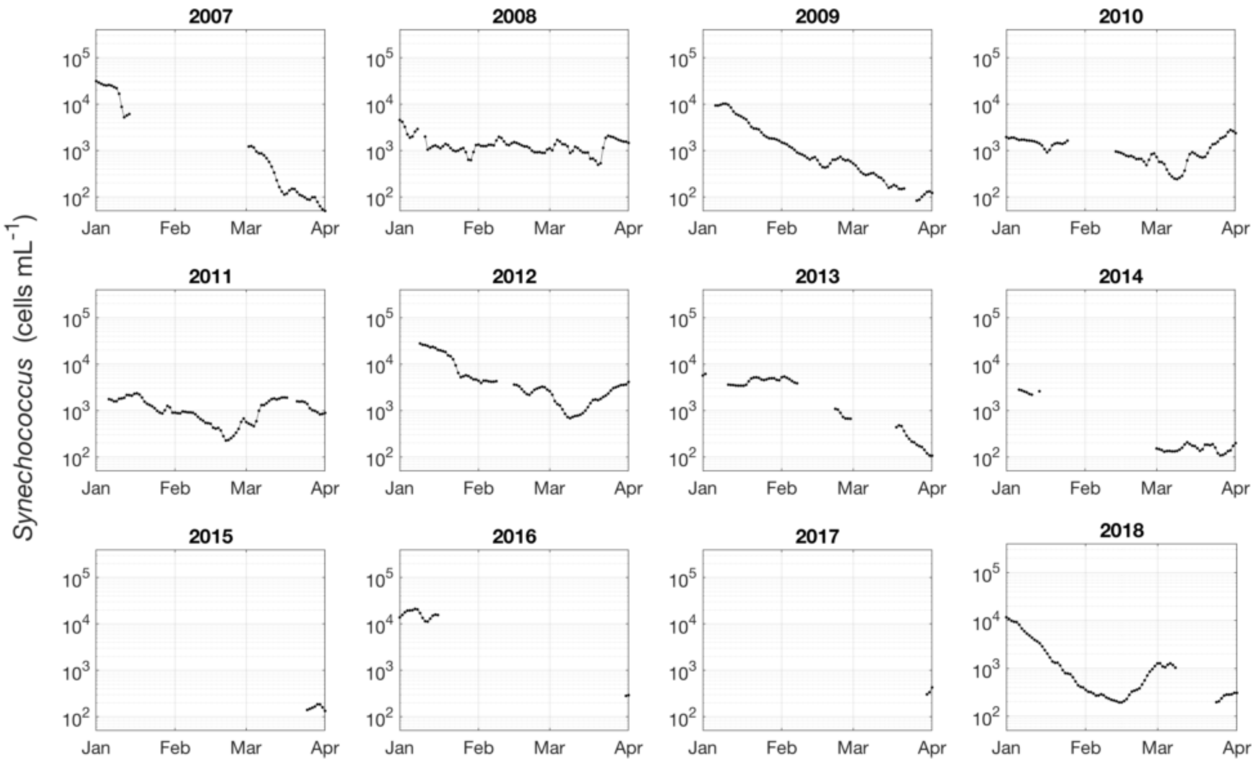

Figure S10: *Synechococcus* cell concentration during winter months for each year in dataset, log scale. Note that data is not available for years 2003-2006 during this season, and limited data in 2015, 2017.

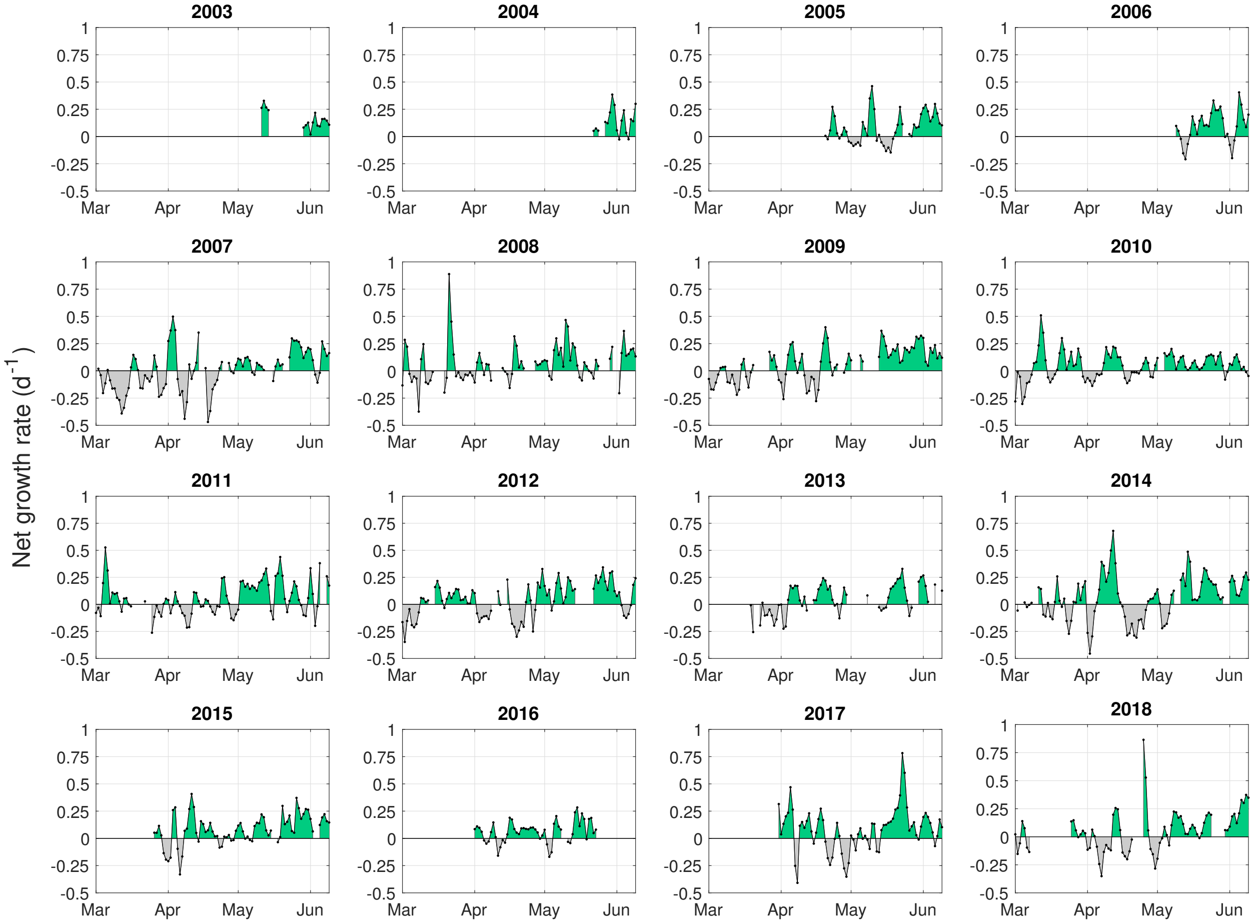

Figure S11: Daily net growth rate for each year in dataset from March - mid-June (black line). For daily continuous data, green shaded areas indicate positive net growth rate, while gray indicates negative net growth rate.

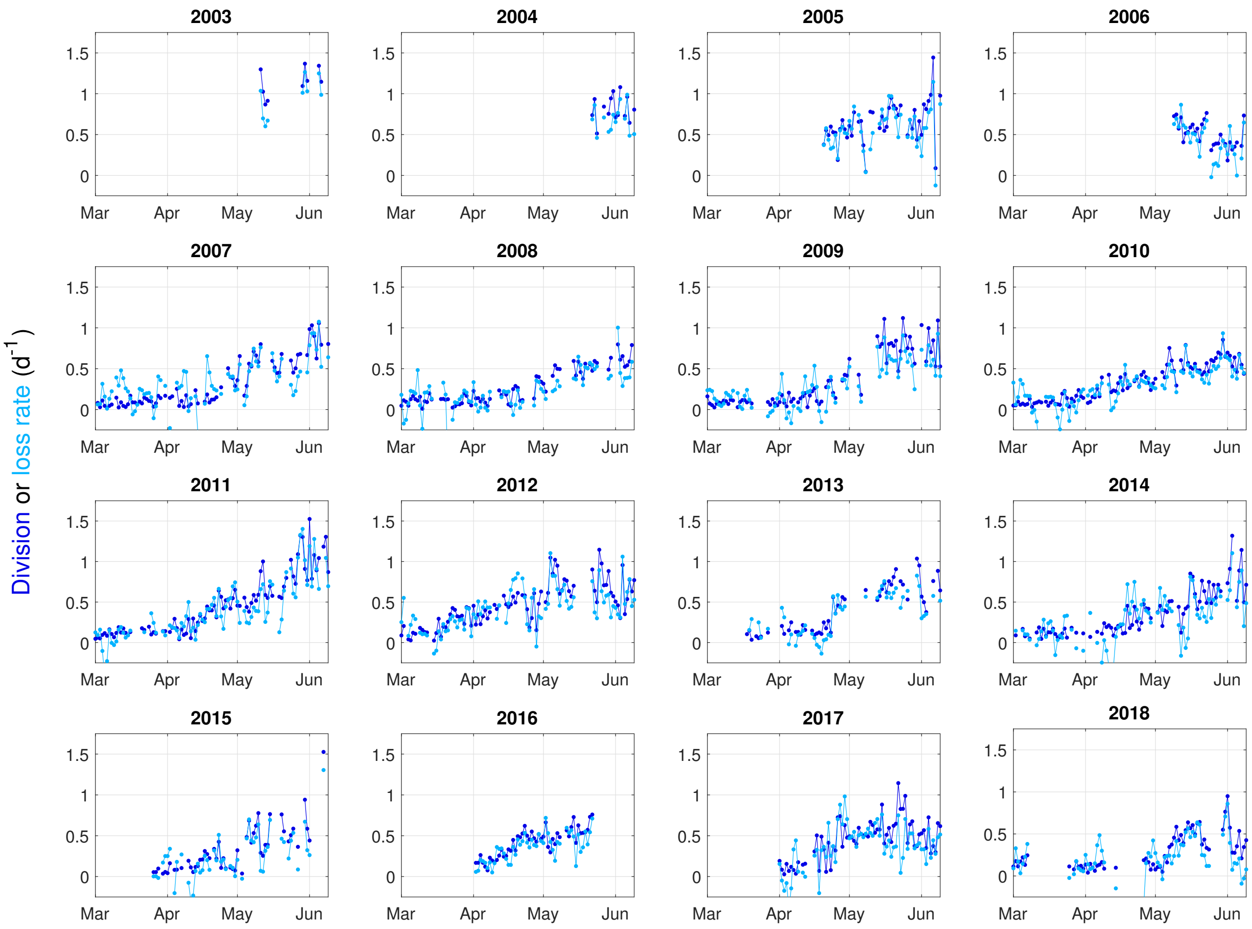

Figure S12: Division and loss rate (dark blue, light blue markers respectively) for each year in dataset from March - mid-June. Markers are connected by lines for daily continuous data.

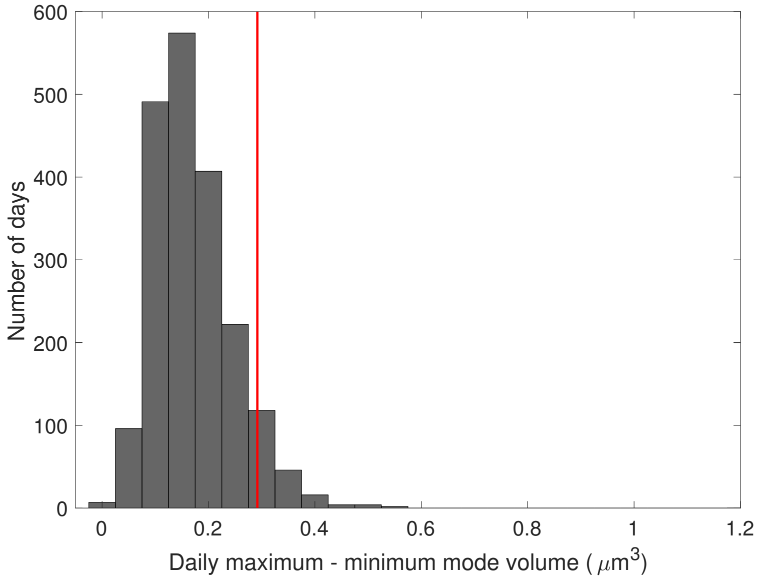

Figure S13: Distribution of differences between daily minimum and maximum mode volume for each day in dataset. Red line indicates annual volume change of minimum cell volume.

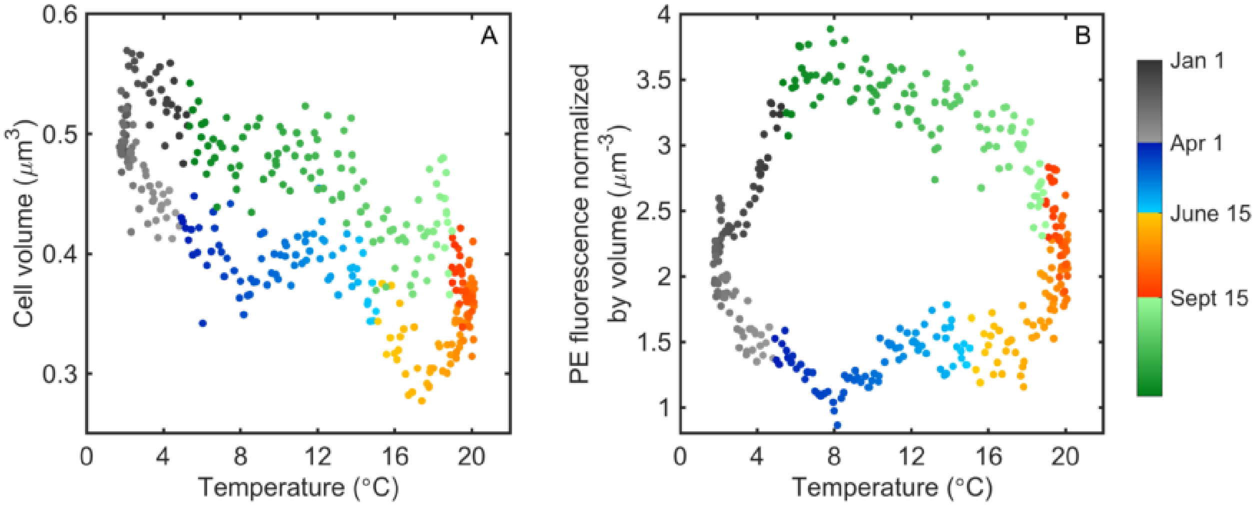

Figure S14: Relationship between climatologies of temperature and A) cell volume and B) cellular PE normalized by cell volume. Color indicates season and year day.

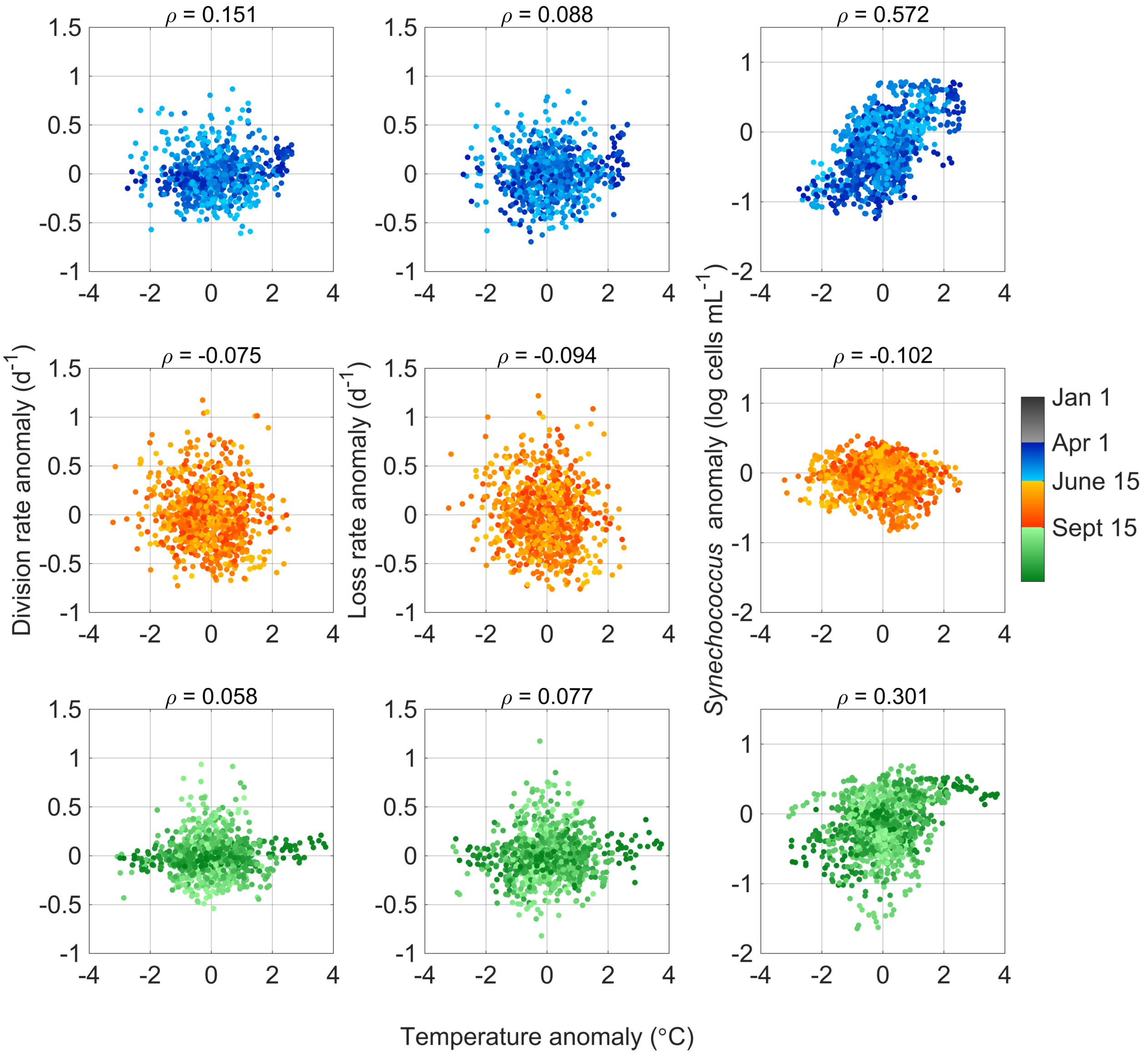

Figure S15: Relationship between daily *Synechococcus* anomalies and daily temperature anomalies for spring (top panels), summer (middle panels) and fall (bottom panels) for division rate anomaly (left panels), loss rate anomaly (middle panels), and *Synechococcus* concentration anomaly (right panels). Color indicates year day. Pearson's correlation coefficient (ρ) between anomalies is indicated above each plot.

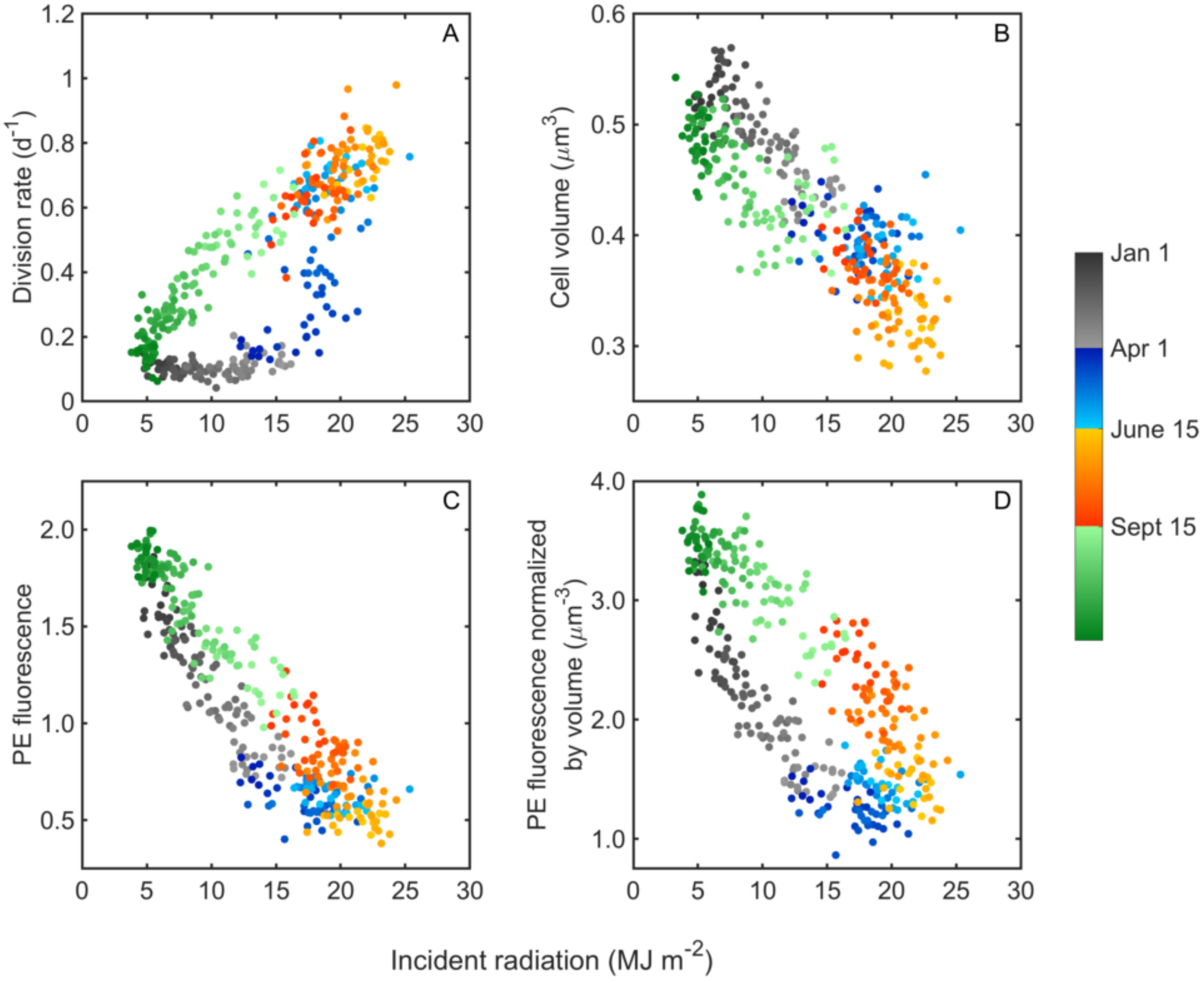

Figure S16: Relationships between climatology of incident radiation and climatologies of A) division rate, B) cell volume, C) PE fluorescence and D) cellular PE fluorescence normalized to cell volume. Color denotes season and year day.

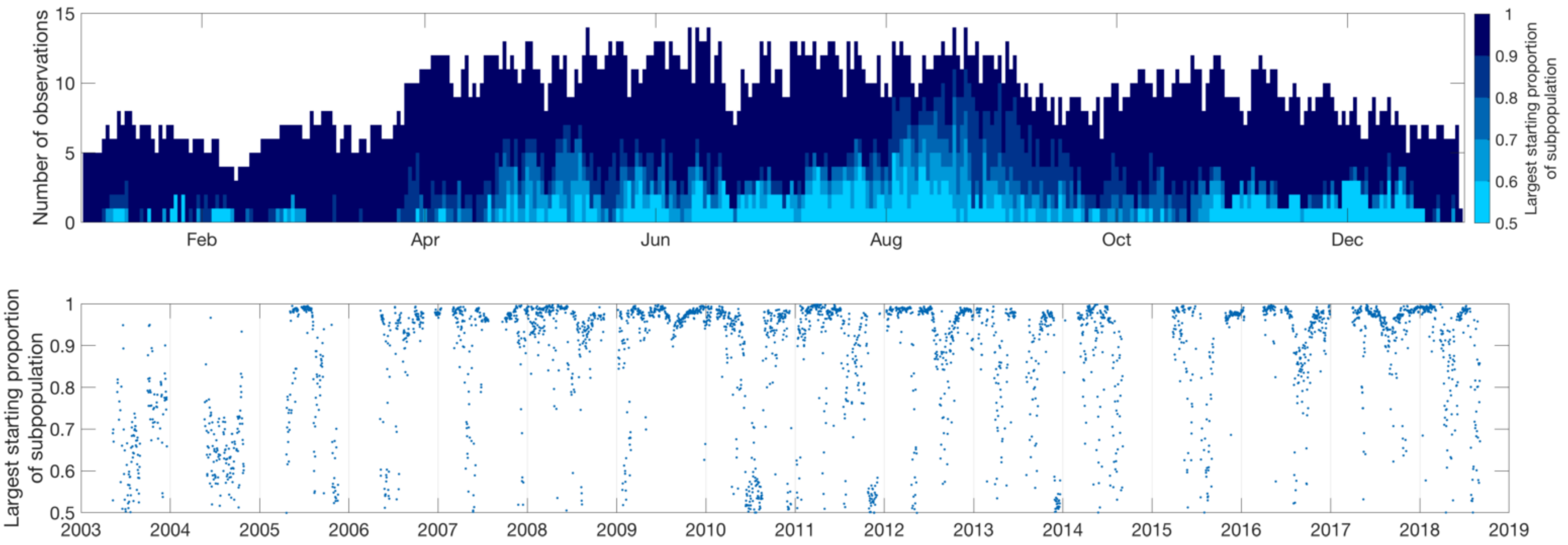

Figure S17: Top panel: distribution of year days for which model estimates are available, color coded by largest (of two) starting proportion for a subpopulation. Bottom panel: time series of largest starting proportion of subpopulation. Note that for many days, one population seems to be sufficient (i.e. values are > 0.9).
